# A review of microbes mediated biosynthesis of silver nanoparticles and their enhanced antimicrobial activities

**DOI:** 10.1016/j.heliyon.2024.e32333

**Published:** 2024-06-04

**Authors:** Chhangte Vanlalveni, Vanlalhruaii Ralte, Hlawncheu Zohmingliana, Shikhasmita Das, Jasha Momo H. Anal, Samuel Lallianrawna, Samuel Lalthazuala Rokhum

**Affiliations:** a Department of Botany, Mizoram University, Tanhril, Aizawl, Mizoram 796001, India; b Department of Botany, Pachhunga University College, Aizawl, 796001, Mizoram, India; c Department of Chemistry, National Institute of Technology Silchar, Silchar, 788010, India; d Natural Products and Medicinal Chemistry Division, CSIR - Indian Institute of Integrative Medicine, Jammu, 180001, India; e Department of Chemistry, Govt. Zirtiri Residential Science College, Aizawl, 796001, Mizoram, India

**Keywords:** Green synthesis, Silver nanoparticles, Microbes-assisted synthesis, Antimicrobial activities, Extracellular, Intracellular

## Abstract

In recent decades, biosynthesis of metal and (or) metal oxide nanoparticles using microbes is accepted as one of the most sustainable, cost-effective, robust, and green processes as it does not encompass the usage of largely hazardous chemicals. Accordingly, numerous simple, inexpensive, and environmentally friendly approaches for the biosynthesis of silver nanoparticles (AgNPs) were reported using microbes avoiding conventional (chemical) methods. This comprehensive review detailed an advance made in recent years in the microbes-mediated biosynthesis of AgNPs and evaluation of their antimicrobial activities covering the literature from 2015-till date. It also aimed at elaborating the possible effect of the different phytochemicals, their concentrations, extraction temperature, extraction solvent, pH, reaction time, reaction temperature, and concentration of precursor on the shape, size, and stability of the synthesized AgNPs. In addition, while trying to understand the antimicrobial activities against targeted pathogenic microbes the probable mechanism of the interaction of produced AgNPs with the cell wall of targeted microbes that led to the cell's reputed and death have also been detailed. Lastly, this review detailed the shape and size-dependent antimicrobial activities of the microbes-mediated AgNPs and their enhanced antimicrobial activities by synergetic interaction with known commercially available antibiotic drugs.

## Graphical abstract

**Figure:**
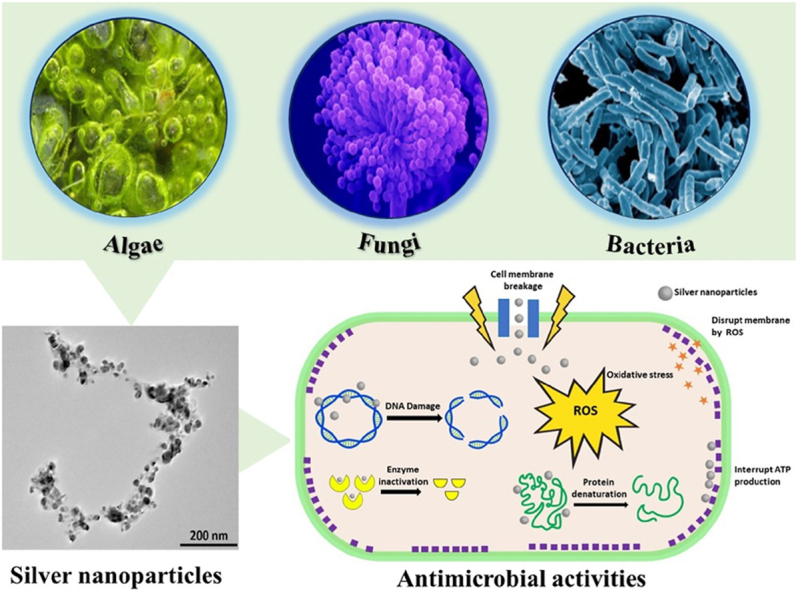

## 1 Introduction

In recent decades, nanotechnology and nanoparticles (nanometer-sized materials, <100 nm) have attracted massive attention owing to their wide applications in numerous fields such as anticancer [1], antimicrobial [2,3], electrochemistry [4], catalysis [5,6], sensors [7], cosmetics [8], and food preservation [9], etc. Compared to bulk materials, nanoparticles (NPs) are known to have several outstanding physical as well as chemical characteristics as a result of their large surface area, high dispersion in solution, etc. Numerous synthesis strategies such as biological, chemical, and physical methods have been reported to produce NPs. Traditional synthesis methods for nanoparticles involve various physical and chemical processes that can be expensive and environmentally harmful due to the release of toxic chemical waste into the environment. Hence, throughout the last few decades, the demand for sustainable, green, and cost-effective alternatives to the conventional synthesis of metal and (or) metal oxide NP production has rapidly increased.

Plant and microbe-mediated synthesis of silver nanoparticles (AgNPs) is an intriguing and ecologically benign strategy that uses plants and microbe's natural capacities to produce nanoparticles with potential applications in various industries. This synthesis process has many benefits, including the utilization of non-toxic and renewable materials, gentle reaction conditions, and the ability to produce silver nanoparticles on a big scale. Plant-mediated synthesis involves using plant extracts as reducing agents to convert silver ions (Ag^+^) into silver nanoparticles (AgNPs) whereas microbe-mediated AgNP synthesis involves microorganisms, including bacteria and fungi which are capable of reducing metal ions to nanoparticles due to the presence of enzymes and other biomolecules [10,11].

**Fig. 1:**
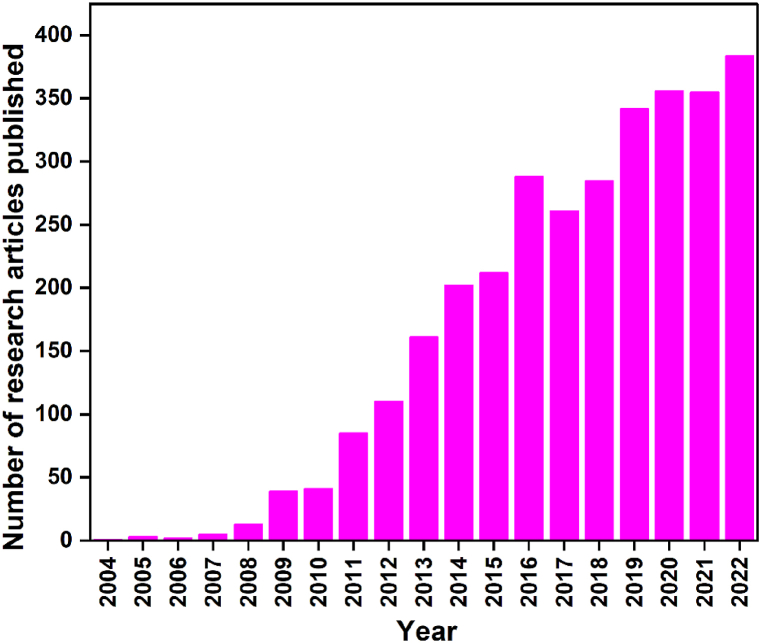
Research papers published annually for the biosynthesis of silver nanoparticles between 2004 and 2022 (data gathered from the Scopus Database).

Biosynthesis of AgNPs is a simple procedure where the biomolecules present in microbes and plants are harnessed to catalyze the formation of AgNPs from different salts of silver (such as AgNO_3_, AgCl, etc.) avoiding the use of harmful chemicals. Owing to this environment's friendliness and simple procedure, the green approach to producing AgNPs is widely pursued. In this context, both microbes and plants have drawn significant attention and have been widely explored in recent years for the green synthesis of AgNPs. The literature survey shown in Fig. 1 revealed the increasing trend of research papers published in the realm of biogenic AgNP synthesis. Data were collected in 2023 from “The Scopus Database” using the keyword “Biosynthesis of silver nanoparticles”. It has expanded dramatically from one research paper published in 2004 to 384 publications in 2022. The increasing number of research paper publications on the biosynthesis of AgNPs indicates a growing interest in this field.

However, it has been found that small AgNPs can lead to lower cell viability [12]. This toxicity issue arises during the preparation and handling of AgNP. During the preparation and handling of AgNP, there is a potential for immediate toxicity and this toxicity can be attributed to the release of Ag ions from smaller AgNPs due to their high surface energy. This release of Ag ions can have detrimental effects on cell viability. These toxic effects may include decreased cell viability and potential damage to DNA, oxidative stress, and impairment of enzyme expression. Additionally, the toxicity of AgNPs can vary depending on factors such as nanoparticle size, shape, concentration, and agglomeration or aggregation. Thus, it is crucial to handle AgNPs with caution and implement proper safety measures to minimize the risk of immediate toxicity during their preparation and handling [13,14].

**Fig. 2:**
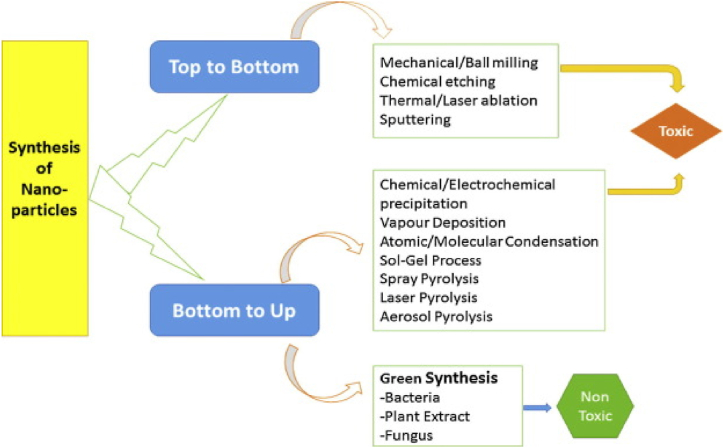
Different methods for the synthesis of nanoparticles. Reproduced with permission from Ref. [3], Copyright 2016, Elsevier.

Two approaches such as “top to bottom” or “bottom to up” were mainly followed for the synthesis of NPs as depicted in Fig. 2. In the former one, a bulk material breaks down into smaller fine nano-sized particles using a variety of methods including milling, grinding, laser ablation and sputtering whereas in latter NPs were obtained by self-assembly of atoms to new nuclei which grow into a particle of nanoscale using chemical and biological methods.

**Fig. 3:**
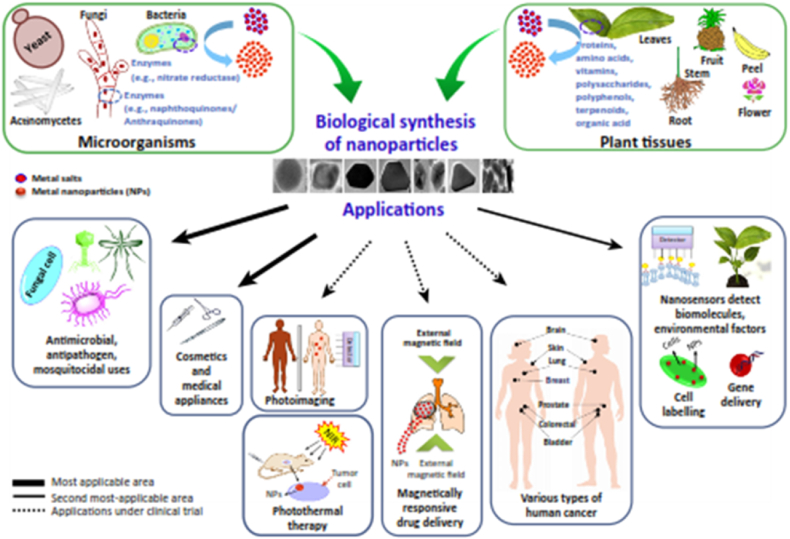
Biological synthesis of NPs and their wide applications in different fields. Reproduced with permission from Ref. [16], Copyright 2016, Elsevier.

To develop green and eco-friendly methods, several works have reported the synthesis of numerous metal oxides and metal nanoparticles such as Ag, Cu, Au, Cr, Co, Ni, Zn, O, etc. using plant extracts and were utilized for different purposes as shown in Fig. 3 [15,16]. Among all these NPs, synthesis, and applications of silver nanoparticles (AgNPs) have been widely examined owing to their high antimicrobial and anticancer activity [17]. More recently, besides the well-known plant-mediated green synthesis, the potential of different microbes such as fungi, bacteria, algae, etc. in the biological production of AgNPs has been widely investigated. The enzymes and biomolecules in the microbial cells are adept at catalyzing the reduction of Ag^+^ to Ag° during the biosynthesis process [18].

Antimicrobial resistance, characterized by the ability of microorganisms to withstand the effects of drugs designed to eliminate them, has evolved into a difficult adversary on the global stage. The antimicrobial resistance (AMR) crisis refers to the escalating worldwide occurrence of infectious diseases in humans that are no longer responsive to any currently available antimicrobial treatment. The consequences of this crisis extend far beyond the confines of individual healthcare systems, transcending borders and impacting communities worldwide. According to recent reports from the World Health Organization (WHO), antimicrobial resistance is not merely a theoretical concern; it is an alarming reality, with estimations suggesting that, if unabated, it could lead to millions of deaths each year [19,20]. Amidst the escalating threat posed by antimicrobial resistance, the international community has mobilized in a collective effort to confront this challenge head-on. However, the question that beckons elucidation is: Why this relentless global search for new antimicrobials? The answer lies in the precarious position we find ourselves in – standing at the precipice of a post-antibiotic era. Existing antimicrobial agents, once hailed as medical marvels, are losing their efficacy at an alarming rate. The emergence of multidrug-resistant strains further worsens the situation, leaving clinicians with limited therapeutic options [21].

Through a comprehensive examination of recent developments, challenges, and potential avenues for intervention, this review aims to contribute to the collective knowledge surrounding antimicrobial resistance.

## 2 Overview of the biosynthesis of AgNPs

### 2.1 Mechanism of AgNP synthesis

**Fig. 4:**
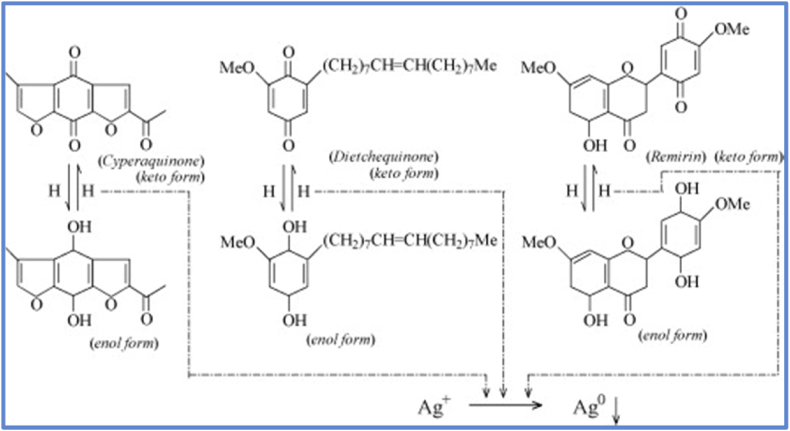
Probable reaction pathways of green synthesis of silver nanoparticles using mesophytes. Reproduced with permission from Ref. [22], Copyright 2009, Elsevier.

A significant number of organic phytochemicals such as proteins, carbohydrates, polyols, phenols, terpenoids, alkaloids, amino acids, enzymes, flavonoids, glycosides, etc. present in the biological entities (plants and microbes) are capable of reducing Ag^+^ ions to metallic silver (Ag°) NPs. In general, for plant-mediated AgNP synthesis, electrons for reduction of Ag^+^ ions to metallic AgNPs are supposed to be derived from keto to enol conversions (Fig. 4), or dehydrogenation of alcohols (catechol) and acids (ascorbic acid) [22]. In the same line, microbial cellular and extracellular oxidoreductase enzymes can do similar reduction processes. Fig. 4 depicts that the process of nano transformation in mesophytes has resulted due to the tautomerization of quinones. *Cyperus* sp., a possible mesophytic genus, has been shown to contain all three forms of benzoquinones, namely cyperoquinone (type I), dietchequinone (type II), and remain (type III). A rapid transformation by the plant extract suggests redial tautomerization. No pH shift was observed or induced in the extract, but gentle warming and subsequent incubation might have activated the quinone congeners resulting in particle size reduction and coalescent cluster formation. Salinity is the most significant environmental constraint on plant growth and productivity. Excessive amounts of ROS (reactive oxygen species) cause oxidative damage to biological molecules, aging, and cell death. The antioxidative system is important for the maintenance of intracellular ROS at appropriate levels. Dehydroascorbate (DHA) reductase (DHAR) catalyzes the re-reduction of DHA to ascorbate. Under alkaline conditions catechol (a dihydric Phenol) can get transformed into protocatechaldehyde and finally into protocatechuic acid. In both cases, reactive hydrogen gets liberated which then participates in the synthesis of silver nanoparticles.

### 2.2 Mechanism of antibacterial activity of silver nanoparticles

**Fig. 5:**
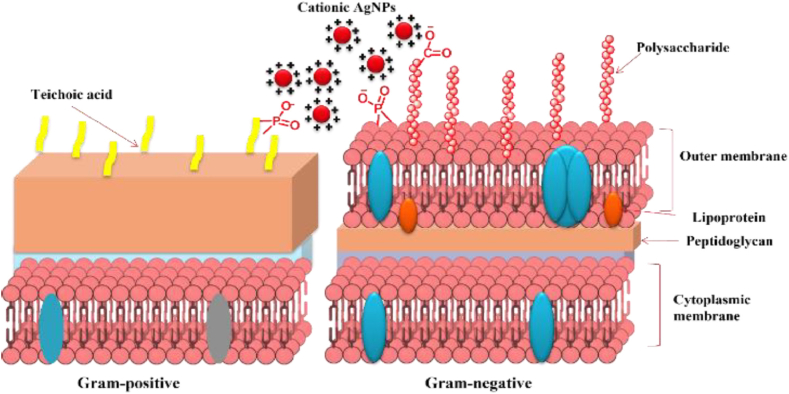
The interaction of AgNPs with Gram-positive and Gram-negative bacterial cell walls is depicted schematically. The positively charged/less negatively charged AgNPs interact electrostatically with the negatively charged bacterial cell wall/membrane and disrupt it. Reproduced with permission from Ref. [30], Copyright 2022, American Chemical chemSociety.

Even though the precise mechanism underlying AgNPs antibacterial activity is still not fully known, however, several potential antibacterial actions have been suggested [23]. AgNPs released silver ions, which are believed to be responsible for killing the microbes. Sulfur proteins can attach themselves to the cytoplasmic membrane and the cell wall due to their electrostatic attraction and affinity for silver ions [23]. The connected ions increased the permeability of the cytoplasmic membrane, causing the bacterial cell to rupture. Successively, after the uptake of free silver ions into cells, respiratory enzymes can be deactivated, generating reactive oxygen species (ROS), and interrupting adenosine triphosphate production. ROS are considered to be a primary cause of cell membrane rupture and deoxyribonucleic acid (DNA) alteration [24,25]. Sulfur and phosphorus, being crucial components of DNA can have detrimental effects on cell reproduction, and DNA replication and can even induce cell death due to the importance of sulfur and phosphorus as parts of DNA, their interaction with silver ions can seriously impair DNA replication, impair cell growth, and even induce cell death. Additionally, by denaturing ribosomes in the cytoplasm, silver ions produced by AgNPs can prevent the synthesis of proteins [26]. In addition to their ability to release silver ions, AgNPs can kill bacteria on their own once they aggregate and stick to the cell surface, causing pits to form on the wall. Based on the cell walls, bacteria are classified into two groups such as Gram-positive and Gram-negative (Fig. 5). Gram-positive bacteria have a thick peptidoglycan layer whereas Gram-negative bacteria have an outer lipid membrane and a thin peptidoglycan layer. Several attachment routes are induced by the cell wall components for the synthesis of NPs. AgNPs can bind to cell walls and membranes, thereby changing their permeability, transport activity, and composition. Hence, most of the AgNPs show higher antibacterial activity against Gram-negative bacteria than Gram-positive bacteria due to their thinner cell walls and less peptidoglycan [[27], [28], [29]]. Furthermore, the negative charge of lipopolysaccharides (LPS) in Gram-negative bacteria eases the adhesion of nanoparticles [30].

### 2.3 Microbes-mediated synthesis

Intra and extracellular synthesis of various forms of AgNPs were reported using several microbes such as bacteria, algae, and fungi. Although intra- and extracellular synthesis of NPs is reported, extracellular synthesis of NPs makes processing and biomass handling stress-free, and hence are most widely investigated [31]. Algae utilize cellular reductase for AgNP synthesis [32], whereas fungal and bacterial species are known to utilize specific reductase enzymes and proteins [33]. Different fungi such as *Candida utilis* (*C.utilis*) [34], *Fusarium oxysporum* (*F. oxysporum*) [35], *Lentinus squarrosulus* (*L. squarrosulus*) [36], *Pleurotus cornucopiae* (*P. cornucopiae*) var*. citrinopileatus* [37], were reported to produce spherical shape AgNPs with 2.78–80 nm. While other fungi such as *Talaromyces purpurogenus* (*T. purpurogenus*) [38], *Phomopsis helianthi* (*P. helianthin*) [39], *Beauveria bassiana* (*B. bassiana*) [40], *Botryococcus braunii* (*B. braunii*) [41], *Penicillium italicum* (*P. italicum*) [42] synthesized AgNPs of a mixture of different shapes viz. oval, triangular, cubical, platelet-like, triangular, hexagonal, quasi-spherical, and rod-shape. The antibacterial properties and activities of fungal-mediated AgNPs against numerous pathogenic and drug-resistant microorganisms were found to be highly effective.

Similarly, numerous algal strains were used to produce AgNPs via biosynthesis. Literature review revealed the synthesis of spherical AgNPs using *Sargassum longifolium* (*S. longifolium*) [43], *Chlorella pyrenoidosa* (*C. pyrenoidosa*) [44], *Spirulina platensis* (*S. platensis*) [45], *Bacillus amyloliquefaciens* (*B. amyloliquefaciens*) [46], *Nostoc linckia* (*N. linckia*) [47]. The formation of AgNPs was confirmed by a characteristic surface plasmon resonance (SPR) peak at 435 nm in the UV–Vis absorbance, signifying the reduction of silver nitrate into AgNPs [47]. The reduction of silver ions to metallic silver Ag° NPs was confirmed by the change in color from pale yellow to brownish. The synthesized AgNPs were assessed for their antimicrobial activities against several microbes such as *Bacillus subtillis* (*B. subtilis*)*, Proteus vulgaris* (*P. vulgaris*)*, Klebsiella pneumoniae* (*K. pneumonia*)*, Pseudomonas aeruginosa* (*P. aeruginosa*)*, Escherichia coli* (*E. coli*)*, Staphylococcus aureus* (*S. aureus* subsp. *Aureus), Staphylococcus epidermidis* (*S. epidermidis), Streptococcus pneumoniae* (*S. pneumonia*)*, Candida albicans* (*C. albicans*)*, Aeromonas hydrophila* (*A. hydrophila*)*, Aspergillus niger* (*A. niger*), etc, and shown modest to high activities. In addition, intra- and (or) extracellular extracts of many bacteria were reported for the synthesis of green AgNPs. *Corynebacterium glutamicum* (*C. glutamicum*) [48], *Mycobacterium bovis* (*M. bovicus*) [49], *Pseudomonas veronii* (*P. veronii*) AS41G [50], *Lactobacillus acidophilus* (*L. acidophilus*) [51], *Bacillus safensis* (*B. safensis*) LAU 13 [52], *B. safensis* [53], and *Delftia* sp. strain KCM-006 [24] etc. are reported to catalyzed the reduction of Ag^+^ ions to metallic AgNPs. There is a specific reason for mentioning the strain number when referring to the biosynthesis of silver nanoparticles using fungi. The strain number typically refers to a specific strain or isolate of a microorganism. Different strains of microorganisms have varying abilities to produce nanoparticles. By specifying the strain number, researchers can provide vital information for others to replicate the experiment under the same conditions. Using a strain number helps avoid confusion between these different strains.

**Fig. 6:**
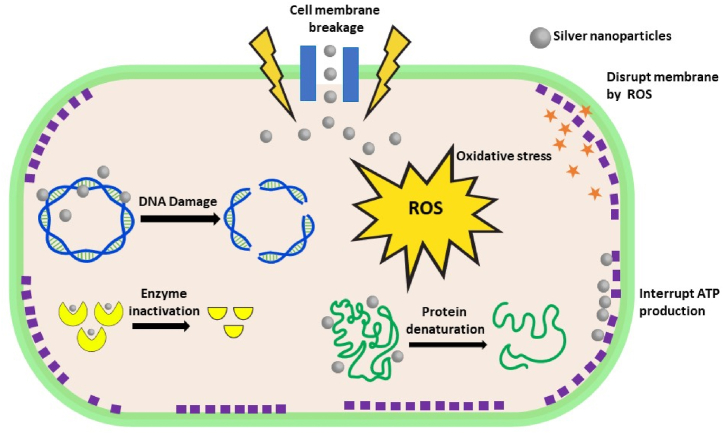
Plausible mechanism of antimicrobial activity of AgNPs.

Of particular interest, the AgNPs synthesized using a cell-free supernatant of *Delftia* sp. strain KCM-006 [24] in conjugation with Miconazole exhibit a synergistic effect resulting in strong fungicidal action, substantial suppression of ergosterol production, and biofilm inhibition by increasing ROS levels. The antifungal activity of AgNPs, miconazole, and miconazole AgNPs was assessed, and it was found that AgNPs showed a moderate decrease in biofilm biomass (20–50 %), whereas Miconazole showed a smaller amount reduction (14–17 %). In the case of Miconazole-AgNPs treated biofilms, the reduction rate was very high (48–64 %), which is 3–4 fold higher as compared to Miconazole alone [24]. Noteworthily, *M. resistens* mediated AgNPs as compared to gold nanoparticles showed higher antimicrobial activities against tested microbes such as *Salmonella enterica* (*S. enterica*)*, Vibrio parahaemolyticus* (*V. parahaemolyticus*)*, Staphylococcus aureus* (*S. aureus*)*, Bacillus anthracis* (*B. anthracis*)*, Bacillus cereus* (*B. cereus*)*, E. coli,* and *Candida albicans* (*C. albicans*) [54]. The precise mechanism of the bactericidal effect of AgNPs has yet not been elucidated and is a topic of hot debate. The antimicrobial activity of AgNPs is widely believed to follow three main routes such as 1) intracellular penetration and damage 2) cell wall and membrane damage, and 3) oxidative stress. Among these, oxidative stress in which the formation of reactive oxygen species (ROS) such as super-oxide (O_2_^−^), peroxy (RCOO·), hydroxyl (·OH), and hydrogen peroxide (H_2_O_2_) provide the main toxicity mechanism against micro-organisms as revealed by Electron Spin Resonance (ESR) [25]. These free reactive oxygen species destroy the cell membrane and penetrate the bacterial cell which results in the denaturation of protein and due course inhibits the growth of microbes (Fig. 6).

**Fig. 7:**
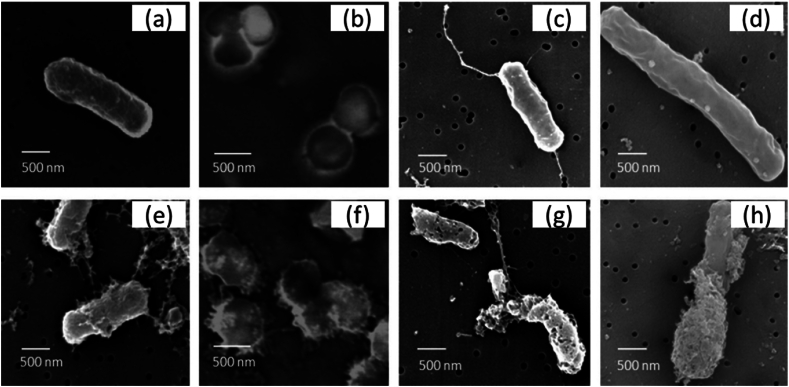
FE-SEM analysis of bacteria treated and untreated with AgNPs: morphology of *B. cereus*, *S. aureus*, *E. coli,* and *P. aeruginosa* (a–d); bacterial cells treated with AgNPs showed membrane damage after 60 min of treatment (e–h). Reproduced from Ref. [55], (Creative Commons CC-BY–NC–ND), Copyright 2017, Elsevier.

Scanning Electron Microscopy (SEM) was a powerful tool to visualize the morphological changes in the microbial cells after treatment with AgNPs. Gopinath et al. [55] used an extracellular extract of the bacteria *Pseudomonas putida* (*P. putida*) for the biosynthesis of AgNPs and verified its antibacterial activity against different bacterial strains. From SEM analysis, they observed that control cells (without AgNPs treatment) exhibited regular and damage-free cells Fig. 7 **a-d**. The four bacterial strains (*S. aureus, B. cereus, P. aeruginosa*, and *E. coli*) treated with AgNPs, showed enormous changes in morphology after 60 min of incubation. They observed a loss of membrane integrity of AgNPs treated bacteria. In the case of *P. aeruginosa* the flagella, which were observed in untreated cells, completely disappeared after the AgNP treatment. About all of the cells displayed rumples and had lost their original morphological structure after 60 min of incubation Fig. 7 **e-h**.

### 2.4 Plants-mediated synthesis

Due to environmental concerns over the use of hazardous chemicals in the synthesis process, researchers are looking for an alternative green route to produce NPs using plant extract [56,57]. Accordingly, extracts of different parts of plants for example leaves, roots, flowers, fruit peels, rhizomes, etc., have been fruitfully explored to produce green AgNPs and were recently reviewed [3,15,58].

## 3 Microbes-mediated green synthesis of AgNPs as antimicrobials

### 3.1 Synthesis using fungi

Table 1 summarizes the biosynthesis of silver nanoparticles using fungi. The biosynthesis of silver nanoparticles (AgNPs) using fungi is an environmentally benign and biologically mediated approach that harnesses the reducing and stabilizing capabilities of fungal components. Fungi are abundant in bioactive compounds such as enzymes, proteins, and polysaccharides that reduce silver ions (Ag^+^) from silver salt solutions into nanoscale silver particles. Spherical AgNPs of size ranging from 20 to 80 nm were biosynthesized using *C. utilis* extracellularly by Waghmare et al. [34]. It showed antibacterial activity against *E. coli, S. aureus,* and *P. aeruginosa*. The antibacterial activity of the synthesized nanoparticles was due to damage to the cytoplasmic membrane. In the recent past, Husseiny et al. [35] studied the biosynthesis of size-controlled AgNPs by *F.oxysporum* and also their antibacterial and antitumor activities. They reported the emergence of spherical, evenly scattered nanoparticles between 5 and 13 nm in size. The greatest zone of inhibition for antibacterial activity against *S. aureus* and *E. coli* was 2 mm and 1.6 mm, respectively, at 80 L of AgNPs. Cytotoxic activity was quantified by an IC50 value of 121.23 cm-^3^. Similarly, AgNPs were synthesized using a heteropolysaccharide (PS) isolated from *L. squarrosulus* (Mont.) Singer [36]. The average diameter of the nanoparticles was 2.78 ± 1.47 nm and mostly spherical. The evaluation of the AgNPs-PS conjugate's antibacterial activity against (MAR) *E. coli* revealed that the bacterium's death (ROS) was caused by the generation of reactive oxygen species. AgNPs have shown a synergistic impact to completely limit bacterial growth when combined with each of the four antibiotics (ampicillin, azithromycin, kanamycin, and netilmicin) to which *E. coli* MREC33 was resistant.

**Table 1:** Biosynthesized AgNPs using fungi.

Sl. No	Fungi	Mode of synthesis	Shape/Size	Test microorganisms	Ref.
1.	*Candida utilis*	Extracellular or Intracellular	Spherical/20–80 nm	*P. aeruginosa, S. aureus, E. coli*	[34]
2.	*Fusarium oxysporum*	Extracellular	Spherical/5–13 nm	*S. aureus, E.coli*	[35]
3.	*Lentinus squarrosulus*	Extracellular	Spherical/2.78 ± 1.47 nm	*E. coli*	[36]
4.	*Pleurotus cornucopiae* var*. citrinopileatus*	Extracellular	Spherical/<100 nm	*C. bruseii,C. albicans,C. glabrata, C. pseudotropicalis*	[37]
5.	*Aspergillus fumigatus*	Intracellular	Spherical/<100	*P. aeruginosa, S. typhimurium, E. coli, Listeria monocytogenes* (*L. monocytogenes*)*, Aspergillus fumigatus* (*A. fumigatus*)*, S. aureus, A. flavus, A. terreus, A. niger, B. cereus, E. faecalis*	[59]
6.	*Penicillium oxalicum*	Extracellular	Spherical/13−23 nm	*A. flavus, P. albicans, A. niger, Aspergillus luchuensis* (*A. luchuensis*)	[60]
7.	*Phoma* spp.	Extracellular	Spherical/106–151 nm	*C. albicans, P. aeruginosa, S. aureus, E. coli, Salmonella choleraesuis* (*S. choleraesuis*)*, A. niger*	[61]
8.	*Fusarium oxysporum*	Extracellular	Spherical/93 ± 11 nm	*A. flavus, A. melleus, A. nomius, A. ochraceus, A. parasiticus*	[62]
9.	*Chlorococcum humicola*	Extracellular	Spherical/12.83 nm	*K. pneumoniae, S. typhi, E.coli, F. solani, F. moniliforme Penicillium* sp*.* (MTCC6489)	[63]
10.	*Chlorella vulgaris*	Extracellular	Spherical/10.69 nm	*S. typhi, K. pneumoniae, E. coli, F. solani, F. moniliforme Penicillium* sp*.* (MTCC6489).	[63]
11.	*Penicillium chrysogenum, Aspergillus oryzae*	Extracellular	Spherical/19–60 nm	(*T. rubrum*)	[64]
12.	*Aspergillus* sp.	Extracellular	Spherical/40–80 nm	*B. cereus, P. putida, E. coli, K. pneumoniae*	[65]
13.	*Aspergillus clavatus*	Extracellular	Spherical/10–25 nm	*C. albicans*, *Pseudomonas fluorescens (P. fluorescens), E.coli*	[66]
14.	*Saccharomyces cerevisiae*	Extracellular	Spherical/5–20 nm	*C. albicans*	[67]
15.	*Guignardia mangiferae*	Extracellular	Spherical/5–30 nm	*E. coli, K. pneumoniae, P. aeruginosa, E. faecalis, Proteus mirabilis* (*P. mirabilis*)*, A. niger, Colletotrichum* sp*., C. lunata, Fusarium* sp*. , Rhizoctonia solani* (*R. solani*)*, S. aureus, B. subtilis, S. epidermidis*	[68]
16.	*Penicillium atramentosum*	Extracellular	Spherical/5–25 nm	*S. aureus, Micrococcus luteus* (*M. luteus*)*, Aeromonas hydrophila* (*A. hydrophila*)*, Enterobacter aerogenes* (*E. aerogenes*)*, B. cereus, Salmonella Typhimurium* (*S. typhimurium)*	[69]
17.	*Alternaria* sp.	Extracellular	Spherical/4–30 nm	*S. aureus, E. coli, S. marcescens, B. subtilis,*	[70]
18.	*Macrophomina phaseolina*	Extracellular	Spherical/5–40 nm	*A. tumefaciens, E. coli*	[71]
19.	*Fusarium chlamydosporum, Penicillium chrysogenum*	Extracellular	Spherical/6 and 26 nm	*A. flavus, A. ochraceus*	[72]
20.	*Ganoderma sessiliforme*	Extracellular	Spherical/45 nm	*B. subtilis, E. coli, L. innocua, M. luteus, S. faecalis*	[73]
21.	*Aspergillus fumigatus*	Extracellular	Spherical/10–34 nm	*B. mycoides, C. albicans, E. coli*	[74]
22.	*Penicillium citreonigrum*	Extracellular	Spherical/6–26 nm	*E. coli, S. aureus, P. aeruginosa*	[75]
23.	*Scopulariopsis brumptii*	Extracellular	Spherical/4.24–23.2 nm	*S. aureus, P. aeruginosa, E. coli*	[75]
24.	*Fusarium oxysporum*	Intracellular	Spherical/1–50 nm	*P. aeruginosa, E. coli*	[76]
25.	*Penicillium oxalicum*	Extracellular	Spherical/10–40 nm.	*S. aureus, E. coli, S. typhimurium*	[77]
26.	*Cladosporium cladosporioides*	Extracellular	Spherical/30–60 nm	*B. subtilis, S. aureus, E. coli, S. epidermis, C. albicans*	[78]
27.	*Pleurotus giganteus*	Extracellular	Spherical/2–20 nm	*E. coli, B. subtilis, P. aeruginosa, S. aureus*	[79]
28.	*Bionectria ochroleuca*	Extracellular	Spherical/4–35 nm	*C. glabrata, S. aureus, C. albicans, E. coli, Candida parapsilosis* (*C. parapsilosis*)	[80]
29.	*Ganoderma lucidum*	Extracellular	Spherical/9–21 nm	*E. coli, S. aureus, C. albicans, Enterococcus hirae* (*E. hirae*)*, L. pneumophila* subsp*. pneumophila, B. cereus, P. aeruginosa*	[81]
30.	Yeast	Extracellular	Spherical/13.8 nm	*E. coli*	[82]
31.	*Penicillium oxalicum*	Extracellular	Spherical/60–80 nm	*S. aureus, S. dysenteriae. S. typhi*	[83]
32.	*Aspergillus flavus*	Extracellular	Spherical/12.5 ± 5.1 nm	*C. glabrata, S. aureus, E. coli, B. subtilis, C. tropicalis, C. parapsilosis, C. albicans, P. aeruginosa*	[84]
33.	*Saccharomyces cerevisiae* (Baker's yeast)	Extracellular	Spherical/3–60 nm	*E. coli, B. subtilis, S. aureus, P. aeruginosa, C. albicans*	[85]
34.	*Pichia fermentans*	Extracellular	Rectangular	*P. aeruginosa, E. coli, Salmonella* sp*., A. tereus, Scedosporium* sp*., C. tropicalis, Fusarium* sp*, S. aureus*	[86]
35.	*Talaromyces purpurogenus*	Extracellular	Spherical, hexagonal, rod-shaped and triangular/4–41 nm	*E. coli, S. Epidermidis*	[38]
36.	*Phomopsis helianthi*	Extracellular	Spherical, pentagonal, hexagonal/35.05 nm	*E. coli, P. aeruginosa*	[39]
37.	*Talaromyces purpureogenus*	Extracellular	Spherical, triangular/25 nm	*B. cereus, E. coli, S. aureus, S. enterica, P. aeruginosa,*	[87]
38.	*Fusarium scirpi*	Extracellular	Quasi-spherical/2–20 nm	*E. coli*	[88]
39.	*Beauveria bassiana*	Extracellular	Triangular, circular, hexagonal/10–50 nm	*E. coli, P. aeruginosa, S. aureus*	[40]
40.	*Botryococcus braunii*	Extracellular	cubical, spherical, and truncated triangular/40–100 nm	*E. coli* (MTCC 442), *K. pneumoniae* (MTCC 109), *F. oxysporum* (MTCC 2087), *S. aureus* (MTCC 96), *P. aeruginosa* (MTCC 441)	[41]
41.	*Rhodotorula* sp*. strain ATL72*	Extracellular	spherical or oval/8.8 and 21.4 nm	*Bacillus* sp*., Staphylococcus* sp*., Shigella* sp*., P. aeruginosa, Streptococcus* sp*., Klebsiella* sp.*, Candida* sp*., E. coli*	[89]
42.	*Penicillium italicum*	Extracellular	Spherical and platelet-like/32–100 nm	*E. coli, C. tropicalis, C. albicans, S. aureus*	[42]

In another work, Owaid et al. [37] reported the biosynthesis of AgNPs utilizing the hot water extract of fresh basidiocarps of *Pleurotus cornucopiae* var*. citrinopileatus*, an eatable fungus with therapeutic qualities. Spherical AgNPs having particle size <100 nm severely inhibited growth of examined Candida species {*Candida glabrata* (*C. glabrata*)*, Candida albicans* (*C. albicans*)*, Candida pseudotropicalis* (*C. pseudotropicalis*)*, Candida krusei* (*C. krusei*)}. Aspergillus fumigatus-synthesized AgNPs inhibited seven bacterial species but not pathogenic fungi (*A. flavus, A. terreus, A. niger, A. fumigatus*). The AgNPs and chitosan nanocomposite exhibited significantly greater antimicrobial activity against all selected microorganisms compared to bare AgNPs [59]. In addition, a biobased extracellular synthesis of spherical AgNPs employing *Penicillium oxalicum* (*P. oxalicum*) was reported. The produced AgNPs exhibited excellent antifungal activity against *A. flavus*, *P. albicans*, *Aspergillus luchuensis* (*A. luchuensis*) and *A. niger* [60].

Extracellular biosynthesis of AgNPs by *Paraboeremia putaminum* (*P. putaminum*) and *P. capsulatum* was reported by Rai et al. [61]. The synthesized nanoparticles, capped with proteins, were spherical and polydisperse. Their sizes ranged from 10 to 80 nm, 5–80 nm, and 5–90 nm for *Planococcus citri* (*P. citri*)*, P. putaminum, and P. capsulatum*, respectively, with average sizes of 31.85 nm, 25.43 nm, and 23.29 nm. Potential antimicrobial activity was tested against *C. albicans, A. niger, E. coli, P. aeruginosa, S. aureus,* and *Salmonella enterica* (*S. choleraesuis*). AgNPs synthesized from *P. citri* demonstrated the lowest minimal inhibitory concentration (MIC) of 0.85 μg mL-1 against *S. choleraesuis.* However, AgNPs synthesized from *P. capsulatum* showed the highest MIC (10.62 μg mL^−1^) against *S. choleraesuis, E. coli,* and *P. aeruginosa*. Evaluation of the antifungal activity of biogenic AgNPs produced by using cell-free filtrate from *F. oxysporum* and its antifungal activity against three toxigenic species belonging to the genera *Aspergillus* section *Flavi* (*A. nomius, A. flavus* and *A. parasiticus*) and two of section Circumdati {*Aspergillus ochraceus* (*A. ochraceus*) and *Aspergillus melleus* (*A. melleus*)} was carried out. The MIC_50_ of the AgNPs against *A. flavus*, *A. nomius,* and *A. parasiticus* was 8 μg mL^−1^, while the MIC_50_ was 4 μg mL^−1^ against *A. melleus* and *A. ochraceus* [62]. Recently, spherical AgNPs were synthesized using *Clavulina humicola* (*C. humicola*) and *C. vulgaris* (*Chlorella vulgaris*), yielding average sizes of 12.83 nm and 10.69 nm, respectively. These synthesized NPs exhibited inhibitory effects against pathogens including *Klebsiella pneumoniae* (*K. pneumonia*), *Salmonella Typhi* (*S. typhi*)*, E. coli, Fusarium solani* (*F. solani*), *Fusarium moniliforme* (*F. moniliforme*), and *Penicillium* sp. MTCC6489 [63].

**Fig. 8:**
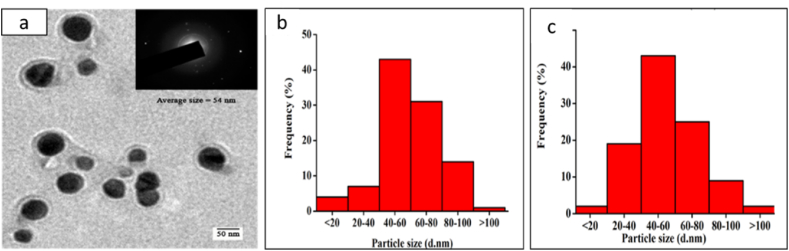
(a) Typical transmission electron micrograph of sphere-shaped AgNPs. Inset showing a single nanoparticle SAED pattern. Particle size distribution histogram of silver nanoparticles as determined using (b) TEM and (c) DLS measurements, reproduced from Ref. [65] (Creative Common Attribution License) Copyright 2015, Public Libra of Science.

Extracellular biosynthesis of AgNPs by *Penicillium chrysogenum* (*P. chrysogenum*) and *Aspergillus oryzae* (*A. oryzae*) and to investigate the antifungal effect of chemically vs biologically synthesized AgNPs comparing with conventional antifungal drugs against *Trichophyton rubrum* was reported by Pereira et al. [64] Chemically synthesized AgNPs (Chem-AgNPs) coated with polyvinylpyrrolidone (PVP) were spherical with an average size of 52 nm and biologically synthesized AgNPs (Bio-AgNPs) were spherical and 19–60 nm in size. All strains of *Trichophyton rubrum* (*T. rubrum*) were resistant to the antifungal effects of chem-AgNPs, whereas bio-AgNPs created by the fungal cell-free filtrate of *Penicillium chrysogenum* (*P. chrysogenum*) showed an antifungal activity that was superior to fluconazole but inferior to itraconazole, terbinafine, and Chem-AgNPs. Jain et al. [65] demonstrated an economical and environmentally affordable approach for the synthesis of protein-capped AgNPs in an aqueous solvent system. As can be seen from Transmission Electron Microscopy (TEM), the synthesized nanoparticles were sphere-shaped and covered with multi-layered protein shell (Fig. 8a). The SAED pattern (Fig. 8a inset) revealed the crystalline nature of AgNPs, whereas TEM analysis further revealed AgNPs ranged between 40 and 80 nm with an average diameter of 54 ± 8.9 nm (Fig. 8b), which is in close agreement with the values attained by dynamic light scattering (DLS) measurements (Fig. 8c). They further proceed to prepare bare AgNPs to compare the antibacterial efficacy of protein-capped and bare AgNPs. The results demonstrated that bare nanoparticles were more efficient than protein-capped AgNPs with varying antibacterial potential against the tested Gram-positive and negative bacterial species and concluded that the inclusion of protein shells on AgNPs can reduce their bactericidal effects.

Verma et al. [66] conducted the biosynthesis of AgNPs using *Aspergillus clavatus* (*A. clavatus*), an endophytic fungus isolated from sterilized stem tissues of *Azadirachta indica* A. Juss. It was found that the produced AgNPs were extracellular, polydispersed spherical or hexagonal particles with sizes ranging between 10 and 25 nm. Antimicrobial activity was performed using a disc-diffusion method against *C. albicans*, *E. coli,* and *P. fluorescens*. According to the findings, the minimum inhibitory concentration for *C. albicans* was 5.83 μg mL^−1^ on average, and the minimum fungicidal concentration was 9.7 μg mL^−1^. The application of *Saccharomyces cerevisiae* (*S. cerevisiae*) for the synthesis of AgNPs was reported by Niknejad et al. [67]. The synthesized NPs antifungal activity was evaluated against fluconazole-susceptible and fluconazole-resistant isolates of *C. albicans* and found that the antifungal activity against fluconazole-susceptible and fluconazole-resistant *C. albicans* isolates was MIC90 values of 2 and 4 μg mL^−1^, respectively. The TEM results showed that nearly 83.6 % of the particles ranged between 5 and 20 nm in size and were fairly uniform, tiny, and spherical.

**Fig. 9:**
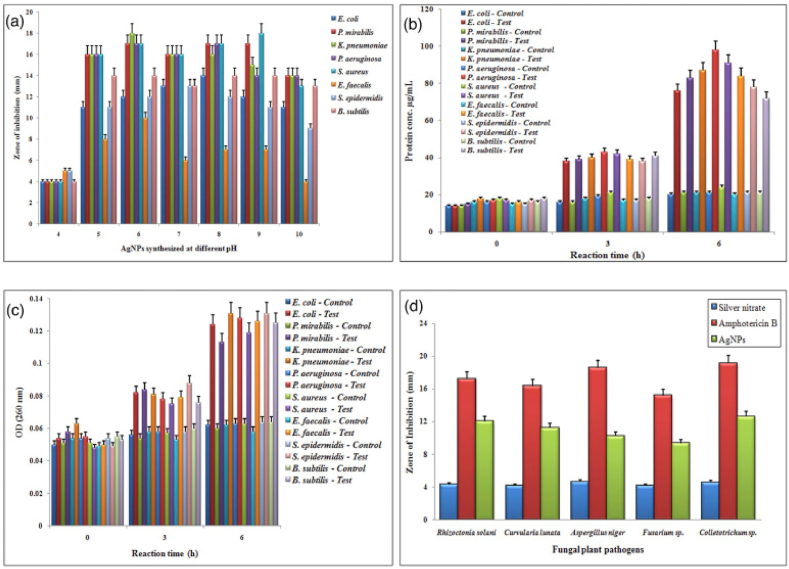
Antibacterial and antifungal activities of *G. mangiferae* mediated AgNPs. Reproduced with permission from Ref. [68], Copyright 2015, Elsevier.

Balakumaran et al. [68] used *G. mangiferae*, an endophytic fungus isolated from medicinal plant leaves, to synthesize spherical-shaped AgNPs with a size range of 5–30 nm. They discovered that the AgNPs destroy the bacterial cells by creating pores, which affect the permeability of the membrane and ultimately result in cell death. In addition, AgNPs showed excellent antibacterial activities against *K. pneumoniae, P. mirabilis, E. coli, S. aureus, S. epidermidis, B. subtilis, E. faecalis, P. aeruginosa,* and antifungal against plant pathogenic fungi such as *Colletotrichum* sp*., C. lunata, A. niger, C. lunata, Fusarium* sp*.* and *Rhizoctonia solani* (*R. solani*) as shown in Fig. 9a–d. The AgNO_3_ and antibiotic Amphotericin B were used to compare the antifungal activities of the synthesized AgNPs, and was revealed to have higher activity compared to silver nitrate solution whereas a lower activity was observed against Amphotericin B (Fig. 9c). Furthermore, the effectiveness of the AgNPs was tested against various cancer cell lines and the cytotoxic effects of AgNPs showed IC_50_ values of 23.84, 27.54 and 63.37 μg mL^−1^ against normal MCF-7 (breast) cells, HeLa (cervical) and African monkey kidney (Vero) respectively, at 24 h incubation period.

Sarsar et al. [69] described the synthesis of biofabricated AgNPs using the filtrate extract of novel fungal strain *P. atramentosum* KM. They found that the protein components of fungal extract are responsible for the reduction of silver nitrate. Antimicrobial activity was shown by the produced SNPs against bacterial strains. SNPs that range in size from 5 to 25 nm showed potent antimicrobial activity against various bacterial species like *B. cereus*, *S. aureus,* and *A. hydrophila.* Sphere-shaped AgNPs with a typical particle size of 4–30 nm were produced from the supernatant of endophytic fungus *Alternaria* sp. isolated from the healthy leaves of *Raphanus sativus* (*R. sativus*). Furthermore, these AgNPs are highly toxic against human pathogenic bacteria for example *E. coli, S. aureus, B. subtilis,* and *S. marcescens*, signifying the possibility of using AgNPs as efficient antibacterial agents [70]. Biosynthesis of AgNPs using cell-free filtrate of phytopathogenic fungus *M. phaseolina* was communicated by Chowdhury et al. [71]. These NPs were discovered to be naturally protein-coated, an 85- kDa protein present in the extracellular solution was responsible for the synthesis and capping of nanoparticles. The particles showed an inhibitory effect on the growth kinetics of multidrug-resistant human bacteria, *E. coli,* and pathogenic plant bacteria, *A. tumefaciens*. In addition to their antimicrobial properties, in vivo and in vitro tests have revealed that AgNPs may also be genotoxic. In the present study, the genotoxicity exhibited by AgNPs was demonstrated by degradation of plasmid post-treatment even with low concentrations of the nanoparticles. A proposed mechanism of DNA damage is through the generation of singlet oxygen.

In order to synthesize AgNPs, Khalil et al. [72] tested the cell-free culture filtrate (CFF) of the fungus *Fusarium chlamydosporum* (*F. chlamydosporum*) and *Penicillium chrysogenum* (*P. chrysogenum*). The synthesized AgNPs were spherical form with a size range between 9 and 17.5 nm for *Penicillium chrysogenum* (*P*. *chrysogenum*) AgNPs (PAgNPs) and from 6 to 26 nm for *Fusarium chlamydosporum* (*F. chlamydosporum*) AgNPs (FAgNPs). In addition, the AgNPs also demonstrated notable antifungal activity and efficacy in thwarting mycotoxin production. Hence, using *A. flavus* as a test microorganism the minimum inhibitory concentration (MIC) was 48, 45, and 50 mg mL^−1^ for FAgNPs, PAgNPs, and the antifungal compound itraconazole, respectively. Also, when testing *Aspergillus ochraceus* (*A. ochraceus*) FAgNPs, PAgNPs, and itraconazole led to MIC values of 51, 47, and 49 mg mL^−1^, respectively. The statistical MIC values to inhibit completely the total aflatoxin production by *A. flavus* were 5.6 and 5.9 mg mL^−1^ for PAgNPs and FAgNPs, respectively, and to inhibit the ochratoxin A production by *A. ochraceus* 6.1 and 6.3 mg mL^−1^ for PAgNPs and FAgNPs, respectively. AgNPs were tested for cytotoxicity on human normal melanocytes (HFB 4) and it revealed a cell survival of 80 % and 75 % at a concentration of 6 mg mL^−1^ for FAgNPs and PAgNPs, respectively. To learn more about the mechanism underlying AgNPs antibacterial activity, the probable leakage of proteins and DNA was studied and the results showed that the presence of AgNPs in the spore suspension caused leakage of proteins as well as DNA. In general, PAgNPs damaged cellular membranes more effectively than FAgNPs. Moreover, *A. flavus* was more susceptible to the leakage of proteins and DNA than *A. ochraceous*. The OD of the proteins leaked from *A. flavus* spore cells incubated with FAgNPs and PAgNPs was 2.05 and 3, respectively, and in the case of DNA leakage, the OD was 1.91 for FAgNPs and 2.27 for PAgNPs. The leakage of proteins from *A. ochraceous* spore cells was greater in the case of PAgNPs (OD 2.86) than for FAgNPs (OD 1.94). DNA leakage brought on by FAgNPs and PAgNPs had OD values of 1.67 and 1.89, respectively. This was attributed to the smaller size of PAgNPs than those of FAgNPs.

The wild mushroom Ganoderma sessiliforme (G. sessiliforme), has been shown to have numerous pharmacological properties including antibacterial, antioxidant, anti-HIV, anti-inflammatory, antiproliferative, antidiabetic, anticancer, antitumor, hypocholesterolemic, and hepatoprotective, was used by Mohanta et al. [73] to demonstrate the synthesis of AgNPs. The potential impacts of AgNPs on food safety and control were evaluated by the antimicrobial activity of the synthesized AgNPs against common food-borne bacteria, namely, *B. subtilis, E. coli, S. faecalis*, *L. innocua,* and *M. luteus*. The findings of this study showed that synthesized AgNPs can be used to control the growth of food-borne pathogens and may have potential applications in the food packaging sector to reduce rapid food spoilage, a major problem that has spread throughout the world due to the obvious involvement of common harmful food-borne bacterial strains. Furthermore, the AgNPs were evaluated for antioxidant activity (DPPH), biocompatibility (L-929, normal fibroblast cells), and cytotoxic effects on human breast adenosarcoma cells (MCF-7 & MDA-MB231) to highpoint their potential for use in a variety of bio-applications.

Othman et al. [74] synthesized AgNPs through fungal mediation of *Aspergillus fumigatus* (*A. fumigatus*). The synthesized AgNPs were sphere-shaped with 90 % of distribution below 84.4 nm which exerted both antimicrobial and antitumor activities against pathogenic microorganisms {*Bacillus mycoides* (*B. mycoides*), *E. coli,* and *C. albicans*). They also investigated its use as an antibacterial finishing agent in textile materials to protect against particular contagious microbes. Their finding revealed that the optimal conditions for the biosynthesis of AgNPs could be attained using 60 % (v/v) of cell-free filtrate (CFF) and 1.5 mM of AgNO_3_ at pH 10.0 after 90 min. The IC_50_ values against in vitro tumor cell lines were found to be 31.1, 33.5, 40.9, and 45.4 μg mL^−1^ for HCT116, MCF7, and PC3, A549, respectively. Hamad et al. [75] worked on the extracellular synthesis of AgNPs by using two filamentous fungi *Scopulariopsis brumptii* Salv.-Duval and *Penicillium citreonigrum* (*P. citreonigrum*) Dierckx. He investigated the antibacterial activity of biosynthesized AgNPs at two concentrations (550.7 and 676.9 mg L^−1^) and interacted with *S. aureus*, *E. coli,* and *P. aeruginosa* for different durations (15, 60, and 120 min) and found out that it has excellent antibacterial property against the tested microorganisms. He also stated that this technology can provide comprehensive antimicrobial solutions for rural populations by using polyurethane foam as a silver carrier and nano-silver solution for the eradication of pathogenic bacteria in polluted water. The synthesized nanoparticles required 60 min to bring the number of all types of bacteria to nil at a concentration of 676.9 mg L^−1^, while the nano-silver coated on foam required 24 h. In two cycles, the nano-silver coating on the foam effectively removed bacteria, but during the third cycle, the effectiveness decreased.

The biological production of AgNPs using *F. oxysporum* was described by Srivastava et al. [76] and in-silico identification was used to determine the antibacterial activity of the nanoparticles using protein-ligand interaction studies. The morphology of the nanoparticles showed that the majority of them were sphere-shaped between the range of 1–50 nm. *P. aeruginosa* and *E. coli* were chosen for insilico research, and metal docking was performed using the licensed software, SYBYL. Five membrane proteins were selected using Uniprot. The ligand docked deeply in the binding pockets of outer membrane proteins (OMPs) of *E. coli* and *P. aeruginosa*. No development of bacterial cells was noticed in the first 5 h at 20, 50, and 100 μg mL^−1^ of Ag NPs. When the concentration of AgNPs was 20 μg mL^−1^, no growth of *E. coli* could be detected at 50 h, indicating that the minimum inhibitory concentration (MIC) of AgNPs to *E. coli* was 20 μg mL^−1^. AgNPs with a concentration of 10 g mL^−1^ and higher worked as a bactericide against *P. aeruginosa*. In-vitro methods using both solid and liquid media further confirmed the outcomes of in-silico studies.

In yet another very interesting work, Rose et al. [77] employed a statistical method response surface methodology (RSM) to optimize the AgNP production using fungi *Penicillium oxalicum* (*P. oxalicum*) GRS-1. The RSM studies revealed that the maximum production of AgNPs with a concentration of 136 ppm was achieved at pH 7.2 with a silver nitrate concentration of 1.975 mM and 86 h using the crude cellular extract of *P. oxalicum* GRS-1. The morphological study of the AgNPs using TEM analysis discovered that the AgNPs were sphere-shaped with sizes ranging from 10 to 40 nm. The biosynthesized nanoparticles showed excellent antibacterial action against common food-borne pathogens such as *E. coli*, *S. aureus,* and *Salmonella Typhimurium* (*S. typhimurium*), with minimum bactericidal concentrations of 16, 32, and 32 g mL^−1^, respectively.

The biogenic synthesis of AgNPs using *Cladosporium cladosporioides* (*C. cladosporioides*) and its antioxidant as well as antimicrobial activity was studied by Hulikere et al. [78] *S. wightii*, a brown alga, was used to isolate *C. cladosporioides*. Its 18 s rDNA sequence was compared to validate its identification. AgNPs have been tested for free radical scavenging activity and antimicrobial activity and the results showed that they have significant antioxidant potential activity and antimicrobial activity and were found to be equally effective against Gram-negative and Gram-positive bacteria as well as the fungus.

Using extracts of the edible wild mushroom *Pisaster giganteus* (*P. giganteus*), Debnath et al. [79] carried out the synthesis of AgNPs and tested the generated AgNPs for antibacterial and amylase inhibitory activities. At a concentration of 100 g mL^−1^ the antibacterial effects of synthesized AgNPs, aqueous AgNO_3_, aqueous mushroom extract, and streptomycin (positive control) on both Gram-negative and Gram-positive bacteria were studied. Biosynthesized AgNPs showed more antibacterial potentiality against Gram-negative bacteria. On test bacterial strain, the aqueous mushroom extract exhibited negligible antibacterial action. Green nanoparticles were reported to have MICs of 12, 10, 14, and 15 g mL^−1^ against *E. coli*, *P. aeruginosa*, *B. subtilis*, and *S. aureus*, respectively. The in vitro anti-diabetic effect of green synthesized AgNPs was investigated by comparing it to acarbose, a common anti-diabetic medication. Biosynthesized AgNPs significantly inhibited the activity of amylase when compared to acarbose, this resulted in the finding that the percentage of amylase inhibition decreased with increasing concentration of green synthesized AgNPs.

Using the extracellular filtrate of the epiphytic fungus *Bionectria ochroleuca* (*B. ochroleuca*), Vishwasrao et al. [80] produced AgNPs that were incorporated into cotton and polyester fabrics. The nanoparticles formed were spherical falling in the range of 8–21 nm. The antimicrobial activity of the AgNPs-loaded fabrics against *S. aureus* and *E. coli*, as well as clinically relevant *C. albicans*, *C. glabrata*, and *Candida parapsilosis* (*C. parapsilosis*), indicated that the AgNPs impregnation of cotton and polyester fabrics was effective. AgNPs effectively prevented *P. aeruginosa* biofilm development while being nontoxic to *Galleria mellonella* (*G. mellonella*) larvae, indicating a viable biotechnological use. *P. aeruginosa* is a Gram-negative, multidrug-resistant bacterium that uses a variety of survival methods, including the biofilm lifestyle. The current research findings indicate that AgNPs were very effective at controlling biofilm development even at low concentrations. The current study findings indicate that biogenic AgNPs are effective against *P. aeruginosa* biofilm development, implying that they could be employed to treat infections caused by Gram-negative pathogenic bacteria. Cotton fabric contains a larger percentage of AgNPs, which effectively inhibits yeast development. Surprisingly, the lower amount of AgNPs impregnated on polyester did not affect its antibacterial efficacy in comparison to cotton fabric. This is an unexpected and surprising outcome for a future scale-up process that might be completed in fewer steps and time, with reduced expenditure in the synthesis of the final antifungal substance. Overall, this technique might save costs while providing effective antibacterial efficacy against yeasts.

Aygün et al. [81] synthesized AgNPs using reishi mushroom, *Ganoderma lucidum* (*G. lucidum*) extract. According to TEM images, AgNPs are sphere-shaped, with a diameter range of 15–22 nm. Furthermore, the antioxidant activity of 1-Diphenyl-2-picrylhydrazyl (DPPH) was also studied. At 250 ml L^−1^, the maximum DPPH scavenging percentage was measured at 76.45 %. According to the DNA cleavage activity data, the green AgNPs caused single-strain DNA cleavage activity for 30 and 60 min at 50 and 100 mg L^−1^. The antibacterial activity of AgNPs was also studied, and the MIC values of AgNPs against *B. cereus*, *Enterococcus hirae* (*E. hirae*), and *S. aureus* were discovered to be 128, 16, and 64 g L^−1^, respectively. The MIC values for gram-negative bacteria were also recorded as 16, 64, and 128 g L^−1^, respectively, against *E. coli*, *L. pneumophila,* and *P. aeruginosa.*

Arya et al. [41] used the extract of green alga *B. braunii* for the synthesis of silver and copper nanoparticles and they found that the size of the nanoparticles produced by algae was in the range of 10–70 nm and 40–100 nm, respectively. The shapes of those NPs were cubical, spherical, and truncated triangular. The two Gram-negative bacterial strains *P. aeruginosa* and *E. coli*, the two Gram-positive bacterial strains *S. aureus* and *K. pneumoniae,* and the fungal strain *F. oxysporum* were all found to be highly toxic to these biosynthesized nanoparticles. The highest ZOI was found against *S. aureus* (22 mm). *K. pneumoniae* (20 mm) had the next-highest zone of inhibition, which was followed by *E. coli* (18 mm) and *P. aeruginosa* (17 mm) and *F. oxysporum* had the lowest zone of inhibition (12 mm). Moreover, the minimum concentration required to stop the growth of the microbes is less in the case of silver as compared to copper. The biosynthesized nanoparticles show the least activity towards the fungus *F. oxysporeum.* Even the biogenically created AgNPs displayed a lower MIC value against both Gram-negative and Gram-positive bacteria than the positive control medication chloramphenicol. For the fungus *F. oxysporum*, Griseofulvin and nystatin were used as the positive control drug.

Soliman et al. [89] studied the biological synthesis of AgNPs with marine pink yeast, *Rhodotorula* sp. Round and oval-shaped nanoparticles were confirmed by UV–visible spectrum analysis, and their sizes ranged from 8.8 to 21.4 nm. By completely inhibiting the growth of a broad variety of Gram-negative and Gram-positive bacteria as well as fungi with low MIC values, the AgNPs demonstrated excellent antibacterial action. Using AgNPs as a treatment and as a control, proteins were taken out from *Candida* sp., *E. coli,* and *Bacillus* sp. at two different separation speeds, and the results of this research revealed various alterations in the protein pattern. At the high-speed separation, these changes were very noticeable, whereas at the low-speed separation, the changes were barely noticeable. In general, samples treated with AgNPs had more new protein bands and increased band intensity than untreated samples. In samples of *E. coli* that were separated at a speed of 4000 rpm, neither the protein profile nor the strength of the bands changed. Following the use of AgNPs to treat *Candida* sp. and *Bacillus* sp., the protein profile underwent qualitative and quantitative modifications, as revealed by the SDS-PAGE analysis. AgNPs capacity to induce new proteins as a stress response may be responsible for the emergence of new bands and the rise in band intensity. While the protein profile is intact in *E. coli* treated with AgNPs, the band intensities were substantially lower than those of the control. This suggests that AgNPs affinity for the thiol groups present in proteins, which causes the protein chain to unfold, may also contribute to the breakdown of the proteins.

**Fig. 10:**
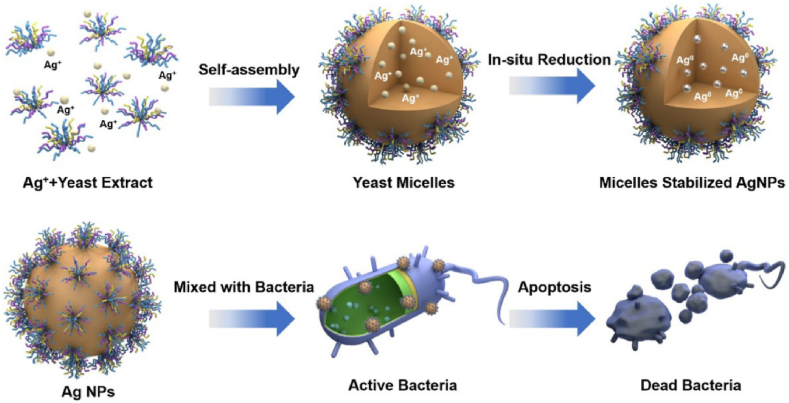
Green synthesis of AgNPs and its antibacterial activity are shown schematically, reproduced from Ref. [82] (Creative Common Attribution International License) Copyright 2020, Springer. (For interpretation of the references to color in this figure legend, the reader is referred to the Web version of this article.)

Taha et al. [42] reported extracellularly biosynthesized spherical and platelets-like AgNPs using *P.italicum* isolated from lemon fruits and its cytotoxic, antioxidant, and antimicrobial activities were examined. The NPs whose size is in the range of 32–100 nm, exhibited shapes that were nearly spherical in some cases while platelet-like in others. Also, the nanoparticles demonstrated strong antimicrobial action against a variety of diseases, such as bacteria and fungi. When coupled with ampicillin, AgNPs consistent spherical shape and small size, with an average size of 13.8 nm, lowered the resistance of ampicillin-resistant *E. coli* cells. It was observed that the AgNPs when treated in combination with ampicillin displayed greater antibacterial efficacy compared to ampicillin alone. NPs having zeta potential values either less than +30 mV or more than −30 mV are considered to be stable. Owing to electron-fixed revulsion, NP aggregation is difficult for elements with a zeta-balancing potential (±30 mV) [90]. The surface charge stability of the synthesized AgNPs was identified by using zeta potential analysis. At a lower pH value of 3, the zeta potential of AgNPs showed a slightly negative charge (−3.2). The zeta potential of AgNPs decreased monotonically from - 12.1 mV at pH 7.0 to −24.4 mV at pH 11.0, confirming that the negatively charged groups were lying on the surface of AgNPs (Fig. 10) [82].

Feroze et al. [83] have done research based on a biogenic synthesis of AgNPs from fungal metabolites of *Penicillium oxalicum* (*P. oxalicum*). The AgNPs had a roughly spherical form and a typical size of 60–80 nm. The antibacterial efficacy of biosynthesized AgNPs was tested against *Salmonella Typhi* (*S. typhi*)*, Shigella dysenteriae* (*S. dysenteriae*), and *S. aureus*. The maximum ZOI recorded against *S. aureus* and *S. dysenteriae* was 17.5 ± 0.5 mm (mm) for both species and 18.3 ± 0.60 mm for *S. typhi* whereas, the normal cell filtrate or fungal biomass of *P. oxalicum* exhibit no zone of inhibition.

In 2022, Fouda et al. [84] reported the synthesis of AgNPs using an extracellular extract of *A. flavus.* The obtained NPs had a typical size of 12.5 ± 5.1 nm and were spherical. The NPs were further examined for their antimicrobial activity against *C. glabrata, B. subtilis, S. aureus, P. aeruginosa, C. albicans, C. parapsilosis, E. coli,* and *C. tropicalis.* In this study, it was found that lowering the AgNP concentrations also reduced their activity. For example, the largest inhibition zones were measured for AgNPs at a concentration of 100 ppm, with a diameter of 18.7 ± 0.5, 17.7 ± 0.6, 20.7 ± 0.6, and 20.8 ± 0.3 mm for *B. subtilis*, *S. aureus*, *P. aeruginosa*, and *E. coli*, respectively. At 50 ppm, the formed inhibition zones were reduced to 15.3 ± 0.5, 14.7 ± 0.6, and 18.5 ± 0.5.

In another recent work, *S. cerevisiae* (Baker's yeast) was used to produce spherical silver nanoparticles in the size range of 3–60 nm. The antimicrobial efficacy was found to be concentration-dependent. With inhibition zones of 21 ± 2.1, 20.9 ± 0.7, 19 ± 0.8, 18 ± 1.4, and 15.6 ± 1.6 mm, respectively, the pathogens *C. albicans*, *P. aeruginosa*, *B. subtilis*, *S. aureus,* and *E. coli* displayed a high level of inhibitory effect at the maximum AgNPs dose (100 g mL^−1^). At a lower concentration (50 g mL^−1^ of AgNPs), however, it demonstrated less antimicrobial activity against the tested strains of *C. albicans*, *B. subtilis*, *P. aeruginosa*, *S. aureus,* and *E. coli*. The corresponding inhibition values were 18 ± 0.8, 16.3 ± 1.2, 15.3 ± 1.2, and 13.1 ± 1.4 mm respectively. Interestingly, Gram-positive bacteria (*C. albicans*) are more susceptible to AgNPs than Gram-negative bacteria (*E. coli*) as can be seen from the ZOI, which is a rare case [85].

Pathogens showing resistance schemes towards AgNPs have been a growing concern in the field of nanotechnology and antimicrobial research. Several studies have reported on the resistance mechanisms employed by pathogens against AgNPs. For instance, research conducted by *Rhizophora apiculata* found that AgNPs synthesized by this organism showed a significantly lower number of bacterial colonies on the agar plate compared to silver nanoparticle-treated cells. This reduction in colony formation is attributed to the smaller size of the nanoparticles and their large surface area, which enhance their ability to increase membrane permeability and induce cell disruption [91]. The emergence and spread of antibiotic-resistant pathogens are mainly due to the misuse and overuse of antibiotics, both in humans and animals. Additionally, factors such as poor infection control practices, inadequate sanitation, and international travel contribute to the spread of resistant pathogens. Understanding the mechanisms and factors influencing antibiotic resistance is crucial for developing effective strategies to combat this issue [92]. Additionally, metal nanoparticles such as silver can bind to the active sites of bacteria, inhibiting their cell cycle function and leading to resistance. Furthermore, silver nanoparticles have been widely used in the medical field, particularly in the development of antibacterial biomedical devices [93].

Bhatnagar et al. [38] utilized an extracellular pigment produced by *Talaromyces purpurogenus* (*T*. *purpurogenus*) to synthesize AgNPs. To achieve the desired low pigment concentration of 0.5 g L^−1^, the optimum precursor concentration and reduction duration were found to be 6 mM and 24 h, respectively, with a particle size distribution of 4–41 nm, the synthesized nanoparticles had spherical, rod-shaped, hexagonal and triangular shapes. The particles were stable (24.8 mV) and polydispersed, according to DLS and zeta potential measurements. Biosynthesized AgNPs had minimal inhibitory values of 32 and 4 g mL^−1^ for *S. epidermidis* and *E. coli*, respectively. Furthermore, using the drug 5-fluorouracil as a positive control, the cytotoxicity of the AgNPs was examined in vitro against the HeLa, HepG2, and HEK-293 cell lines used to study human cervical, liver, and kidney cancer. Significant activity was recorded against the HepG2 cell line with a half-maximal inhibitory concentration of 11.1 μg mL^−1^.

Gond et al. [39] demonstrated a single-step synthesis of AgNPs from cell filtrate of an endophytic fungus isolated from *N. arbortristis*. AgNPs had an average size of 35.05 nm, with smaller AgNPs being spherical and larger AgNPs being pentagonal or hexagonal. In comparison to the AgNO_3_ solution employed as the control, the maximum inhibition zones by AgNPs were 14 mm each against *E. coli* and *P. aeruginosa* (10 mm and 9 mm). According to reports, endophytic *Phomopsis* species produce antimicrobial substances including phomol and antimalarial phomoxanthones.

Although spherical AgNPs were generally obtained in the biosynthesis, other shapes such as rectangular [86], mixed spherical, hexagonal, cubical, rod-shaped, oval, triangular-shape, platelets-like structure [38,39,42,89], were reported. Recently, Chauhan et al. [86] reported the extracellular synthesis of rectangular AgNPs and zinc oxide (ZnO) NPs using *P. fermentans* which were isolated from spoilt fruit pulp. Interestingly, the AgNPs inhibit the growth of tested clinical pathogens for example *P. aeruginosa, E. coli, Salmonella* sp*., S. aureus, C. tropicalis, Scedosporium* sp*., A. tereus, Fusarium* sp*.* whereas ZnO NPs were able to inhibit only *P*. *aeruginosa*, thereby proving the higher antimicrobial activity of AgNPs compare to ZnO NPs. Their research showed that the inhibitory efficacy against clinical infections was increased by the interaction between the conventional antibiotic disc and biosynthesized metallic nanoparticles.

To synthesize AgNPs, Hu et al. [87] used the mycelial extract of the endophytic fungus *T. purpureogenus*. Then they examined the AgNPs for their antibacterial, anti-proliferation, and cell injury healing properties. The nanoparticles had an average size range of 25 nm and the shapes were like triangles and circles. The AgNPs significantly decreased the growth of bacterial pathogens at the least inhibitory concentration of 16.12 g mL^−1^ for Gram-positive bacteria and 13.98 g mL^−1^ for Gram-negative bacteria. Moreover, AgNPs (2 g mL^−1^) demonstrated a potent anti-proliferation activity in A549 cells. Surprisingly, normal NIH3T3 cells were not cytotoxic to AgNPs (Swiss albino mouse embryonic tissue). The NPs also showed a remarkable capacity for cell wound repair. The wound area decreased in a dose-dependent manner, the wound area was 1.976, 1.824, and 1.4364 cm^2^, respectively, at different concentrations (1, 5, and 10 μg mL^−1^) of the experimental group. Furthermore, the wound area of the treated group was smaller than the untreated control area (2.356 cm^2^).

Rodriguez-Serrano et al. [88] synthesized biogenic AgNPs with extracellular metabolites secreted by the soil fungal strain *F. scirpi*, whereas Tyagi et al. [40] focused on the extracellular AgNPs synthesis using Entomopathogenic fungus *Beauveria bassiana* (*B. bassiana*). The generated AgNPs were of various shapes, such as triangular, circular, and hexagonal, and size ranges from 10 to 50 nm. The synthesized nanoparticles were found to be stable under a fungal solution (Zeta: −22 mV). It was discovered that pH 6.0 and temperature 25 °C were the best conditions for the production of nanoparticles. Growth kinetic studies have confirmed that AgNPs have ongoing time-dependent effects on *P. aeruginosa*, *E. coli,* and *S. aureus*. At 30 °C, *E. coli* showed the greatest drop in O. D560 during the 36 h growth investigation, followed by *P. aeruginosa* (63.3 %) and *S. aureus* (56.8 %). The MIC values of fungal-assisted AgNPs against *E. coli*, *P. aeruginosa,* and *S. aureus* were observed to be 2.5, 3, and 4.5 ppm, respectively. Ciprofoxacin's MIC values were found to be 0.5, 0.5, and 0.7 ppm, whereas the combined MICs of AgNPs and Ciprofoxacin were 0.4, 0.4, and 0.5 ppm against *E. coli*, *P. aeruginosa,* and *S. aureus*, respectively. This clearly shows the synergistic effect of AgNPs and Ciprofloxacin.

Danagoudar et al. [94] synthesized AgNPs using the flavonoid-producing endophytic fungus, *Aspergillus austroafricanus* (*A. austroafricanus*) which was isolated from *Zingiber officinale* (*Z. officinale*). AgNPs were spherical and ranged in diameter from 2 to 51.34 nm. The cytotoxic activities were assessed and it was observed that AgNPs significantly outperformed non-cancerous (HEK-293) cells in terms of their cytotoxic ability against cancer (MCF-7, A431, and HepG2) cell lines.

Cui et al. [95] worked on the biosynthesis of AgNPs mediated by *Trichoderma longibrachiatum* (*T.longibranchiatum*)*,* originally isolated from the roots of oak trees using orthogonal experimental design (OED). The created AgNPs showed many functional group moieties and spherical cubic crystal nanoparticles with diameters ranging from 5 to 50 nm. To test the toxicity of AgNPs, mulberry leaves were used to deliver them to *B. mori* larvae. Supplement of AgNP had no detrimental effects on silkworm larval development or cocoon quality, showing that AgNP was safe for silkworms at 50 mg L^−1^. The results will offer important safety information for the widespread use of AgNPs.

### 3.2 Synthesized using algae

Table 2 summarizes the biosynthesis of AgNPs using algae. The use of algae in the biosynthesis of silver nanoparticles (AgNPs) is an ecologically benign and sustainable technology that makes use of the unique biochemical composition of algae. Silver nanoparticles (AgNPs) have been synthesized from algae using a variety of methods. The antibacterial potency of greenly synthesized AgNPs and their low cytotoxicity point to their potential use in biomedical and pharmacological applications [96], as a prototype for future nanomedicine or drug delivery systems [97], as biotechnology antioxidant agents [98], as a promising material against infectious bacteria [99], for both commercial and therapeutic uses [100]. Typically, measurements of UV–vis, FTIR, and X-ray diffraction (XRD) were used to monitor the development of silver nanoparticles. Size and zeta potential measurements using DLS and morphological analysis using TEM were also used to describe the AgNPs [96,99,101]. Fabrication of AgNPs using *S. longifolium* and their microbial activity against several pathogens as reported by Rajeshkumar et al. [43] suggested the pH dependence of the reaction mixture in the AgNP synthesis. The antifungal activity against test organisms was found to increase with increasing concentration of AgNPs.

**Table 2:** Biosynthesized silver nanoparticles from algae.

Sl. No.	Algae	Mode of synthesis	Shape/Size	Test microorganisms	Ref.
1.	*Sargassum longifolium*	Extracellular	Spherical/20–80 nm	*C. albicans, A. fumigatus, Fusarium* sp.	[43]
2.	*Sargassum longifolium*	Intracellular	Spherical/40–85 nm	*C. albicans, Fusarium* sp*., A. fumigatus*	[43]
3.	*Chlorella pyrenoidosa*	Extracellular	Spherical/2–15 nm	*A. hydrophila, K. pneumoniae, Acinetobacter* sp*., S. aureus*	[44]
4.	*Spirulina platensis*	Extracellular	Spherical/30–50 nm	*P. vulgaris, K. pneumoniae, S. aureus, S. epidermidis, B. cereus, E. coli,*	[45]
5.	*Gelidium amansii*	Extracellular	Spherical/27–54 nm	*V. parahaemolyticus, P. aeruginosa, E. coli, A. hydrophila, B. pumilus, S. aureus*	[102]
6.	*Bacillus amyloliquefaciens*	Extracellular	Spherical/100 nm	*P. aeruginosa, E. coli, S. pyogenes, C. albicans, S. aureus*	[46]
7.	*Bacillus subtillis*	Extracellular	Spherical/100 nm	*P. aeruginosa, S. aureus, E. coli, S. pyogenes, C. albicans*	[46]
8.	*Nostoc linckia*	Intracellular	Spherical/5–60 nm	*E. coli, S. aureus* subsp*. aureus, S. pneumoniae, C. albicans, A. niger, B. subtilis, P. aeruginosa*	[47]
9.	*Oscillatoria* sp.	Intracellular	Spherical/10 nm	*S. aureus, P. aeruginosa, Citrobacter* sp.*, S. typhi, B. cereus, E. coli*	[96]
10.	*Gracilaria birdiae*	Extracellular	Spherical/20.3–94.9 nm	*E. coli,S. aureus*	[97]
11.	*Dunaliella salina*	Extracellular	Spherical/35 nm	*E. tobbaci, E. coli*, *B. subtilis*	[103]
12.	*Caulerpa racemosa*	Extracellular	Spherical/5–25 nm	*S. aureus, P. mirabilis*	[104]
13.	*Neodesmus pupukensis*	Extracellular	Spherical/52–179 nm	*C. albicans,E. coli, K. pneumoniae, P. aeruginosa, S. aureus, S. marcescens, A. niger, A. flavus, A. fumigatus, F. solani*	[98]
14.	*Padina* sp.	Intracellular	Spherical/25–60 nm	*S. aureus, P. aeruginosa*	[99]
15.	*Portieria hornemannii*	Extracellular	Spherical/35–50 nm	*V. harveyii, V. alginolyticus, V. anguillarum, V. parahaemolyticus*	[90]
16.	*Chlorella ellipsoidea*	Extracellular	Spherical/220.8 ± 31.3 nm	*K. pneumoniae, P. aeruginosa*	[105]
17.	*Navicula cincta*	Intracellular	Spherical/32 nm	*B. subtilis, S. aureus, E. coli, K. pneumoniae, P. aeruginosa, Shigella flexneri* (*S. flexneri*)*, V. cholera, S. typhi, A. niger, A. fumigatus, R. stolonifer, A. flavus*	[106]
18.	*Champia parvula*	Extracellular	Spherical/79 nm	*S. aureus, S. mutans, C. albicans, E. faecalis*	[107].
19.	*Trichodesmium erythraeum*	Extracellular	Spherical. irregular, cubical/26.5 nm	*V. cholerae, S. aureus, P. mirabilis, S. pneumonia, K. pneumonia, E. coli*	[108]
20.	*Gelidium corneum*	Extracellular	Spherical or angular/20–40 nm	*C. albicans, E. coli*	[100]
21.	*Neochloris oleoabundans*	Extracellular	Quasi-spherical/16.63 nm	*E. coli*	[109]
22.	*Acanthophora specifera*	Intracellular	Cubical/33–81 nm	*S. aureus, Salmonella* spp*., E. coli, C. albicans, B. subtillis*	[110]
23.	*Ulva lactuca*	Extracellular	Cubical/20–35 nm	*P. falciparum*	[111]
24.	*Pithophora oedogonia*	Extracellular	Cubical and hexagonal/25–44 nm	*P. aeruginosa,E. coli, V cholerae, S. flexneri, B. subtilis, M. luteus, S. aureus,*	[112]
25.	*Sargassum muticum*	Extracellular	NA	*E. coli*, *B. subtilis*	[113]

A high degree of consistency in the morphology of the synthesized AgNPs was shown by *Chlorella pyrenoidosa* (*C. pyrenoidosa*) [44]. Aziz et al. reported that the reduction of AgNO_3_ was done after 22 days of initial incubation when the biomolecules had optimal activity. Their study reported that the AgNPs produced have a size range of 2–15 nm with only a small fraction having above 20 nm. The antibacterial efficacy was examined against four pathogenic microorganisms, *K. pneumoniae, Aeromonas hydrophila* (*A. hydrophila*), and *Acinetobacter* sp. The highest ZOI was shown by *A. hydrophila* and *Acinetobacter* sp. Extracellular synthesis employing *S. platensis* aqueous extract was reported by Sharma et al. [45] with the generation of well-dispersed, extremely stable, spherical AgNPs with an average size of 30–50 nm. Here, the synthesized nanoparticles demonstrated strong antibacterial activity against pathogenic Gram-positive, such as *S. aureus*, *S. epidermidis,* and *B. cereus* bacteria, as well as Gram-negative bacteria like *E. coli*, *P. vulgaris,* and *K. pneumoniae*, with a maximum zone of inhibition (ZOI) of 31.3 ± 1.11 in *P. vulgaris.*

A green approach using marine red algae *viz., G. amansii* [102] has also been reported and its antibacterial potentials against pathogenic Gram-positive (*S. aureus,* B. pumilus*)* and Gram-negative bacterial (*E. coli,* V. parahaemolyticus*, P. aeruginosa,* A. hydrophila) pathogens been evaluated. These results imply that the green strategy could successfully reduce bacterial growth by inducing bactericidal action against Gram-positive and Gram-negative pathogens that can form biofilms. Biosynthesis using precursors such as AgNO_3_ by *B. amyloliquefaciens* [46] is also reported where an average diameter smaller than 140 nm is attained. The ZOI was detected on the plates containing AgNPs, when the antibacterial activities against Gram-negative bacteria: *P. aeruginosa, E. coli, Salmonella* sp., as well as Gram-positive: *S. pyogenes, S. aureus,* were evaluated, thus showing their potentiating antibacterial effect. Vanlalveni et al. [47] reported a quick and environmentally friendly way to make AgNPs utilizing the cyanobacterium *N. linckia*, whose diameters were in the 5–60 nm range obtained from TEM. The produced AgNPs demonstrated strong antibacterial activity against two tested fungus strains and four pathogenic bacteria, including *E. coli*, *P. aeruginosa*, and *Staphylococcus aureus* subsp. *aureus* and *B. subtilis*, (*C. albicans* and *A. niger*).

Green route biosynthesis of spherical AgNPs with the size of 10 nm using methanol extract of *Oscillatoria* sp. was reported with functional groups containing hydroxyl, alcohol, phosphate, and amine were found to be responsible for the capping and stability of proteins in the biosynthesis of spherical AgNPs in the size of 10 nm [96]. The inhibition zone for the AgNP's efficient antibacterial activity against the test bacterial pathogens spanned from 1 to 21 mm, and they also displayed significant antibiofilm activity. However, it was found that the toxicity of the AgNPs to *A. salina* (brine shrimp) was negligible. Green synthesis employing a reducing and stabilizing agent i.e., polysaccharide extracted from red algae *Gracilaria birdiae* (*G.birdiae*) [97] has also been reported with favorable characteristics like sphere shape, zeta potential negative, small size, and hydrodynamic diameter ranging between 20.2 nm and 94.9 nm. The stability of the synthesized AgNPs was analyzed during our months and no significant agglomeration was observed. *S. aureus* (Gram-positive) and *E. coli* (Gram-negative) were used to test the AgNPs for antibacterial activity, and all samples displayed antimicrobial activity against *E. coli*.

Recently, an algal *D. salina* assisted production of spherical-shaped AgNPs with an average size of 35 nm was reported. The synthesized NPs exhibit antibacterial activity against three tested microbes such as *B. subtilis*, *E tobbaci,* and *E. coli*. The synthesized silver nanoparticles exhibit similar antibacterial activities against Gram-positive (*B. subtilis*) and Gram-negative (*E. tobbaci*) strains with ZOI of 7 mm each [103]. Synthesis of AgNPs using marine algae i.e., *Caulerpa racemosa* (*C. racemose*) [104] has also been reported. According to the UV–Vis spectrum, the produced AgNPs have a surface plasmon resonance at 413 nm and have demonstrated antibacterial efficacy against human pathogens like *S. aureus* and *P. mirabilis*. Cell-free extracts of *N. pupukensis* [98], a microalga were also employed to synthesize both silver (AgNPs) and gold nanoparticles (AuNPs). The nanoparticles thus formed were screened for their antimicrobial potential and free radical scavenging activity against stable free radicals (2,2-diphenyl-1- picrylhydrazyl). ZOI of 43 mm (*Pseudomonas* sp), 24.5 mm (*E. coli*), 27 mm (*K. pneumoniae*), 39 mm (*S. marcescens*) for AgNPs and ZOI of 27.5 mm (*Pseudomonas* sp) and 28.5 mm (*S. marcescens*) for AuNPs showed promising antibacterial activities against the test organisms [98]. AgNPs antifungal efficacy was further confirmed by the mycelial inhibition rates of 80.6, 57.1, 79.4, 65.4, and 69.8 % against *A. niger*, *A. fumigatus*, *A. flavus*, *F. solani,* and *Candida albicans*, respectively, while AuNPs possessed antifungal potency of 79.4, 44.3, 75.4, 54.9, and 66.4 % respectively.

According to Bhuyar et al. [99], adding marine algae to produce NPs with reasonably homogeneous diameters of 25–60 nm increased AgNP production. It was attained by using an extract from the marine alga *Padina* sp. High antibacterial activity was demonstrated by the produced AgNPs against *P. aeruginosa* and *S. aureus* with ZOI values of 15.17 ± 0.58 mm and 13.33 ± 0.76 mm, respectively. According to their research, the marine alga *Padina* sp. could serve as a substitute source for the manufacture of AgNPs because it produces antimicrobial chemicals that are beneficial against both Gram-negative and Gram-positive bacteria. A red alga was examined for the productive synthesis of AgNPs as an alternative to commercially available antibiotics in the treatment of fish infections caused by *P. hornemannii* [90]. The synthesized were found to be active against the fish pathogens viz., *Vibrio harveyi* (*V. harveyii*), *V. parahaemolyticus*, *Vibrio alginolyticus* (*V. alginolyticus*), and *Vibrio anguillarum* (*V. anguillarum*) with maximum activity against *Vibrio harveyi* (*V. harveyii*) and *V. parahaemolyticus.*

**Fig. 11:**
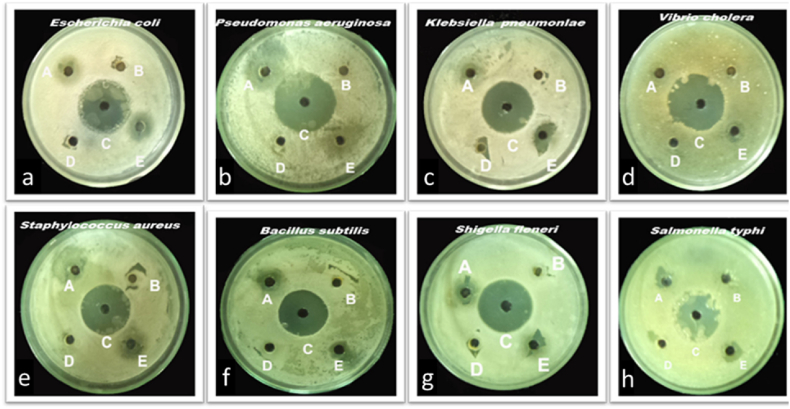
MIC of synthesized AgNPs antibacterial activity against (a) *E. coli* (b) *P. aeruginosa* (c) *K. pneumonia* (d) *V. cholera* (e) *S. aureus* (f) *B. subtilis* (g) *S. flexneri* (h) *S. typhi,* where inside the figures (A) AgNO_3_ (B) algal extracts, (C) chloramphenicol, (D) distilled water, (E) AgNPs. Reproduced from Ref. [106] (Open Access), Copyright 2020, John Wiley & Sons Inc.

Borah et al. [105] employed *C. ellipsoidea*, a green algae for the synthesis of AgNPs. The phytochemicals from the alga, are thought to serve as both reducing and stabilizing agents. Dynamic light scattering reveals that the normal size of the particle is 220.8 ± 31.3 nm, and plasmon resonance at 436 nm indicates the creation of AgNPs. Even after three reduction cycles, the catalytic efficiency was maintained. The AgNPs also demonstrated significant antibacterial efficacy against four selected pathogenic bacterial strains such as *P. aeruginosa, and K. pneumoniae.* Boobalakhrishnan et al. [106] reported for the first time, the synthesis of AgNPs from *N. cincta*, and studied the antimicrobial activity for various pathogenic bacteria and fungi which includes *E. coli, S. aureus, B. subtilis, K. pneumonia, P. aeruginosa, S. flexneri, A. flavus, V. cholera* and *S. typhi,* and*, A. niger, Aspergillus flavus* (*A. fumigatus*) and *Rhizopus stolonifer* (*R. stolonifera*) respectively. A concentration of 5 mM AgNO_3_ of 10 ml mixed with 5 ml of algal extract shows the highest production of AgNPs within 30 under the sunlight. SEM analysis data reveals that the NPs were polydisperse spherical with an average size of 32 nm from dynamic light scattering analysis. The synthesized AgNPs had weak antibacterial activity towards *S. typhi* (12 mm), *K. pneumoniae* (15 mm), *Shigella flexneri* (*S. flexneri*) (13 mm) and *V. cholera* (14 mm) as shown in Fig. 11a–h. The highest antibacterial activity was against *E. coli* (25 mm), *P. aeruginosa* (24 mm), followed by moderate activity against *S. aureus* (22 mm) and *B. subtilis* (20 mm). The AgNPs show a significant ZOI for the tested fungi and a higher ZOI against *A. flavus* (18), *Aspergillus flavus* (*A. fumigatus*) (14), *Rhizopus stolonifer* (*R. stolonifera*) (9) than Nystatin (12), (10), (5) respectively.

Viswnathan et al. [107] recently reported using an aqueous extract of the red seaweed *C. parvula* for the synthesis of AgNPs. The produced AgNPs inhibited all the tested microbes such as *S. aureus, C. albicans*, *S. mutans,* and *E. faecalis;* the highest ZOI was observed in *S. mutans* (ZOI 23 mm). The aqueous extract of *Trichodesmium erythraeum* (*T. erythraeum*) has been used to produce AgNPs in an environmentally benign way, according to Sathishkumar et al. [108]. The synthesized AgNPs were found to be crystalline in nature, irregular, spherical, and cubical with an average size of 26.5 nm. At 500 mg mL^−1^, a radical scavenging experiment revealed antioxidant potential to be 77.01 ± 0.17 % in DPPH, 67.5 ± 0.22 % in Deoxy-ribose, 52.77 ± 0.42 % in ABTS, and 88.12 ± 0.26 % in nitric oxide, respectively. Excellent inhibition against drug-resistant bacterial strains such as *E. coli* (AmikacinR), *S. aureus* (TetracyclineR), and *S. pneumoniae* (PenicillinR) as well as clinical strains (*S. aureus* and *P. mirabilis)* have also been reported from antibacterial studies. *Gelidium corneum* (*G. corneum*)*,* [100] a red alga was also employed for the synthesis of AgNPs whose size ranges between 20 and 50 nm as revealed by TEM micrographs. A high level of antibacterial activity and extremely low MIC values with 0.51 μg mL^−1^ for *C. albicans* yeast and 0.26 μg mL^−1^ for *E. coli* (bacteria) have been revealed from the results of their broth micro dilution test. Prebiofilm and postbiofilm effects were considered in two stages of antibiofilm efficacy studies. Prebiofilm efficacy of 81 % and postbiofilm efficacy of 73.5 % reduction rate in biofilm formation at 0.51 μg mL^−1^ have also been reported from their antibiofilm efficacy studies. By incubating the mixture of AgNO_3_ solution and whole-cell aqueous extracts (WCAEs) of *Neochloris oleoabundans* (*N. oleoabundans*) [109] under light conditions, quasi-spherical AgNPs with an average particle diameter of 16.63 nm were synthesized. The AgNPs were reported to exhibit decent homogeneity as well as antibacterial activities.

Moreover, silver nitrate salt was converted into cubical silver nanoparticles in size ranging from 33 nm to 81 nm via alcohol extraction from *Acanthophora spicifera* (*A. specifera*) [110]. The synthesized AgNPs were reported to significantly reduce the growth of both Gram-negative *Staphylococcus.* spp*., E. coli,* and Gram-positive *S. aureus*, *B. subtilis* in addition to the unicellular fungus *C. albicans*. To create an inexpensive seaweed extract of *Ulva lactuca* (*U. lactuca*), Murugan et al. [111] presented a unique approach of plant-mediated synthesis of silver nanoparticles (AgNPs) as an environmentally friendly tool to combat malaria vectors. The effectiveness of the *U. lactuca* extract and the green-synthesized AgNP against the larvae and pupae of the malaria vector was investigated. *A. stephensi* and their overall findings made it clear that AgNP produced by *U. lactuca* might be used to create new, safer malaria-controlling medications.

In another work, rapid production of AgNPs by reduction of silver nitrate with aqueous extract of *P. oedogonia* having UV–Vis plasmon resonance at 445 nm was also achieved as reported by Sinha et al. [112]. Using scanning electron microscopy (SEM) and dynamic light scattering analysis the size of colloidal AgNPs was determined. The shape of the AgNPs is cubical and hexagonal. The presence of AgNPs was confirmed by energy-dispersive X-ray spectroscopy, which exhibited strong signals in the silver region. Fourier transform infrared spectroscopy of the nanoparticles revealed the presence of protein as a capping agent enclosing the AgNPs. Furthermore, the antibacterial activity of produced nanoparticles demonstrated potential inhibitory efficacy against seven harmful microorganisms examined. Recently, Azwatul et al. [113]. utilized sewage algal (*S. muticum*) bloom for the biosynthesis of silver nanoparticles. As expected, the synthesized AgNPs showed greater inhibition against Gram-negative bacteria (*E. coli*, ZOI 13) than Gram-positive bacteria (*B. subtilis*, ZOI of 9 mm) due to the presence of thick cell walls in Gram-positive bacteria that protect cell breakage and death.

### 3.3 Synthesis using bacteria and cyanobacteria

Water is one of the most common spawning sites for most pathogens. As per the report, in India, 80 % of the diseases are due to bacterial contamination in drinking water thereby requiring the killing of pathogenic microorganisms during water purification, particularly for disinfecting drinking purposes [34]. Table 4 summarizes the biosynthesis of AgNPs using different bacteria and cyanobacteria. Enzymes, proteins, and polysaccharides are just a few of the biomolecules that bacteria and cyanobacteria can produce. These biomolecules can efficiently convert silver ions (Ag^+^) from silver salt solutions into nanoscale silver particles.

Gowramma et al. [48] created AgNPs from a native isolate of *C. glutamicum*. The TEM images confirmed that the typical AgNP size was about 15 nm*.* The spherical and circular morphology was confirmed by Scanning Electron Microscope (SEM). In their study, they showed that the size of the AgNPs can be controlled by controlling the pH. Their study reveals that, at high pH value, the size of the AgNPs becomes smaller while at low pH, the size becomes larger. The nanoparticles varied in size from 45 nm at acidic pH to just around 10 nm at neutral pH. The antimicrobial activity of the nanoparticles was studied against *K. pneumoniae, E. coli, S. flexneri, S. aureus, S. enterica, P. aueroginosa, B. flexus, and B. subtilis* using well diffusion techniques and it was found that the highest antimicrobial activity (ZOI) was found against *K*. *pneumoniae* which was 18 mm in diameter. The zone of inhibition (ZOI) was 16, 15, 15, 14, 12, 8, and 4 mm in diameter for *E. coli*, *S. aureus*, *S. enterica*, *S. flexneri*, *P. aueroginosa,* and *B. subtilis*, respectively.

In another work, Nano-Scaling Silver (NSAg) was synthesized by Abdel-Aziz et al. [49] from *M. bovicus* which was isolated from the soil. The development of a reddish brown color, when M. Bovicus culture supernatant was combined with an equivalent volume of aqueous silver nitrate solution (3 mmolL-1), shows the formation of nanoparticles. These AgNPs was then immobilized on a sodic-montmorillonite clay (MMT1), cetyltrimethylammonium bromide-modified montmorillonite (MMT2), and finally MMT3, which is produced by calcining MMT2 at 300 °C. TEM investigation showed that the NSAgs has a size range of ∼5.4–21.5 nm but the morphology is highly variable. The antimicrobial activity of MMT1, MMT2, and MMT3 was tested by Cup Plate Method and Plate Count Technique on *S. aureus, E. coli,* and *C. albicans* and it was observed that a strong antimicrobial activity was exhibited except by MMT3. The caging of NSAgs, which restricts their release and dissemination across the culture, maybe the cause of MMT3's ineffectiveness.

The intracellular synthesis of AgNPs from the antibacterial of *Rhodococcus* spp. was demonstrated by Otari et al. [114]. After 24 h of incubation, the color changed from colorless to brown, signifying the synthesis of AgNPs. A characteristic peak between 300 and 500 nm due to the surface plasmon resonance (SPR) effect was observed which indicates that the AgNPs were spherical. According to the TEM image of AgNPs, the nanoparticles were between 5 and 50 nm, which is consistent with DLS. Different concentrations of AgNPs are taken (10, 30, and 50 μM mL^−1^ of medium) and used to study their antimicrobial activity against different pathogenic microorganisms. The higher concentration (50 μM mL^−1^) of AgNPs completely arrests the growth of the studied microorganism viz. *E. coli, K. pneumonia, S. aureus* and *Pseudomonas aeruginosa.* Further, AgNPs were prepared by extracellular synthesis of *P. veronii* AS41G by Baker et al. [50]. Under optimized conditions, the synthesis of nanoparticles happened very quickly and it was discovered that alkaline pH had a greater impact on the process than neutral and acidic pH. The TEM microgram shows that the nanoparticles are primarily spherical and range in size from 1 to 50 nm. Some shapes were almost spherical, hexagonal, and triangular. AgNPs were investigated for their antibacterial activity against a variety of bacteria, including *B. subtilis* (MTCC 121), *E. coli* (MTCC 7410), *S. aureus* (MTCC 7443), *K. pneumoniae* (MTCC 7407), *P. aeruginosa* (MTCC 7903), *X. axonopodis* pv. *malvacearum*, *X. oryzae* pv*. oryzae* and *R. solanacearum* and shows significant activity with a clear zone of inhibition. The lowest concentration of AgNPs for inhibition ranges from 31.25 μg mL^−1^ to 250 μg mL^−1^.

Rajesh et al. [51] reported the synthesis of biogenic AgNPs using the Gram-positive bacterium *Lactobacillus acidophilu* (*L. acidophilus*). The bacterium (*L. acidophilus*) functions as both a capping and reducing agent. The TEM micrograph shows that the AgNPs formed have a size range of 4–50 nm. The shape of the synthesized nanoparticles was mostly spherical. The study reported that the leakage of protein and reducing sugar was maximum in 60 μg mL^−1^ after 2 h and 4 h exposure against *K. pneumoniae*. In the recent past, AgNPs were synthesized intracellularly by *S. strains* and tested against several microbes such as *E. coli, S. aureus, S. typhi, K. pneumoniae, P. aeruginosa,* and *P. vulgaris.* The antibacterial activity of biosynthesized AgNPs was highest against *B. cereus* and lowest against *P. vulgaris* [115]. The synthesis of AgNPs was also reported by using *Streptomyces* sp. (09 PBT 005) [116]. The polydispersed nanoparticles have a roughly spherical form and an average size of diameter between 198 and 595 nm which is observed by SEM. Gram-positive and Gram-negative bacteria are both effectively inhibited by the biologically synthesized nanoparticles. The nanoparticles showed good activity against Gram-negative bacteria such as *S. flexneri* and *E. aerogenes* with maximum zones of inhibition (16 mm) at 0.02 M concentration at pH 7.13 mm zones were visible for *K. pneumoniae*, *S. typhimurium*, *S. typhi,* and *P. vulgaris*. Gram-positive bacteria including *S. epidermidis* (15 mm), MRSA (13 mm), and *B. subtilis* (12 mm) had a moderate level of activity.

A. Lateef et al. [52] similarly produced AgNPs by employing keratinase from a strain of *B. safensis* LAU 13. They were spherical with the size ranging from 5 to 30 nm but an average size of ∼8 nm. The study showed that the AgNPs have remarkable activity against five clinical isolates of *E. coli*. For the test strains, the AgNPs produced a maximum ZOI of 12.5 mm and a minimum ZOI of 8.6 mm at a concentration of 150 g mL^−1^. Recently, Singh et al. [117] made use of the *Brevibacterium frigoritolerans* (*B. frigoritolerans*) DC2 strain for the synthesis of silver nanoparticles. The study showed that the average size of the particles was 97 nm and 0.191 polydispersity index. The FE-TEM revealed the spherical shape of the nanoparticles. The antibacterial activity was tested against a pathogenic microorganism including *S. enterica, V. parahaemolyticus, B. cereus, B. anthracis, E. coli,* and *C. albicans* and showed great activity. The ZOI for these microorganisms was in the range of 11 ± 0.2 to 24 ± 1.4 mm diameter for 50 μL of the reaction mixture. The antibacterial activity of many commercial antibiotics, including oleandomycin, lincomycin, novobiocin, penicillin G, vancomycin, and rifampicin was also improved by the AgNPs.

The manufacture of AgNPs was recently carried out by A. Lateef et al. [53] using the cell-free extract of *B. safensis*. The produced AgNPs have a sphere-shaped structure with sizes varying from 5 to 95 nm. Inhibition of *K. granulomatis, S. aureus, E. coli,* and *P. aeruginosa* was achieved at 100 μg mL^−1^. The range of antibacterial improvements of commercially available antibiotics such as cefixime, augmentin, and ofloxacin by Ag-NPs were 20.0–142.9 %, 42.9–114.3 %, and 7.4–54.6 %, respectively. The AgNPs at a final concentration of 5 μg mL^−1^ completely eradicated the population of *P. aeruginosa, S. aureus, A. fumigatus,* and *A. flavus* in the Ag-NPs-treated paint as against the profuse growth seen on the control plates. They also noted that at concentrations of 20–100 g mL^−1^, the synthesized AgNPs exhibited strong DPPH radical scavenging capabilities of 40.56–89.40 %. The high absorbance recorded for the ferric-reducing potential of AgNPs also indicates their antioxidant activities. They also reported that the synthesized AgNPs have different potency of larvicidal activity with 100 % mortality at the end of 12 h.

Similar to this, Kumar et al. [24] created a technique for the manufacture of AgNPs utilizing the cell-free supernatant of the *Delftia* sp. strain KCM-006 and coupled it to the antifit ungal medication miconazole. They have reported the formation of sphere-shaped and monodispersed nanoparticles with an average size of 9.8 nm. The antifungal activity of the AgNPs against several pathogenic *Candida strains* was very good. Significant fungicidal efficacy, suppression of ergosterol production, and biofilm inhibition via raising ROS levels are all displayed by the conjugated AgNPs with miconazole. The antifungal activity of AgNPs, miconazole, and miconazole AgNP was assessed and it was found that AgNP showed a moderate reduction in biofilm biomass (20–50 %), whereas miconazole showed very less reduction (14–17 %). The reduction rate was quite significant (48–64 %) for miconazole–AgNP treated biofilms, which is three to four times greater than for miconazole alone.

**Fig. 12:**
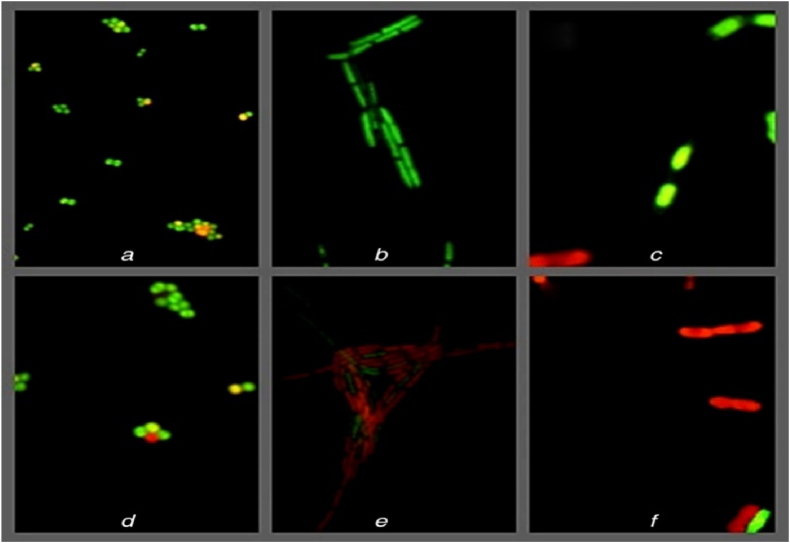
Live/Dead analysis of effective activity of AgNPs on bacterial pathogens control *– (a) S. aureus, (b) B. subtilis, (c) E. coli, Experimental – (d) S. aureus, (e) B. subtilis, (f) E. coli.* Reproduced from Ref. [119], (Open Access), Copyright 2017, Wiley.

The efficiency of the synthesized AgNPs by *B. methylotrophicus* was reported by Wang et al. [118] The synthesized NPs have a spherical shape with 10–30 nm in size. The antibacterial activity was tested by measuring the mean diameter of ZOI against *C. albicans, V. parahemolyticus, E. coli,* and *S. enterica* and it was found that they have a great antibacterial activity compared to standard antibiotics. AgNPs were also synthesized using an acidophilic actinobacterial SH11 strain isolated from pine forest soil which has a spherical and polydispersed shape and with a size in the range of 4–24 nm and an average size of 13.2 (±2.9) nm [119]. The AgNPs exhibited the lowest MIC against *B. subtilis* and *S. aureus* both at 40 μg mL^−1^, followed by *E. coli* at 70 μg mL^−1^. The antibacterial activity of antibiotics was enhanced by AgNPs and tetracycline was the most enhanced one against the tested pathogens as shown in Fig. 12a–f.

In another study, an acidophilic actinobacterial SH11 strain isolated from the mineral horizon of a pure strand of *Picea sitchensis* (Sitka spruce) from the southern end of Hamsterley Forest, County Durham, UK [120], was used for the biosynthesis of AgNPs having a size in the range of 4–45 nm and were mostly sphere-shaped. The antibacterial activity of bio-AgNPs has been investigated against pathogenic bacteria such as *P. aeruginosa, E. coli, K. pneumoniae, S. infantis, S. aureus, P. mirabilis,* and *B. subtilis* using the well-diffusion method. Simultaneously, the synergistic effects of AgNPs with commercial antibiotics against bacterial pathogens have been also investigated using the disc diffusion method. The antibacterial activities of most antibiotics increased in the presence of AgNPs against various tested bacterial pathogenic strains. The combination of AgNPs with tetracycline, kanamycin, ampicillin, and neomycin produced the most notable results against *E. coli*, *S. infantis,* and *K. pneumoniae*, followed by streptomycin and gentamycin.

Recently, Golinska et al. [121] synthesized and investigated the AgNPs from two acidophilic strains of *Pilimelia columellifera* subsp. *pallida*. The antibacterial activity in combination with antibiotics was evaluated against different bacteria. In their study, they demonstrated that when the activity of the antibiotic was increased evaluated in combination with AgNPs made from both actinobacterial strains, the activity of the antibiotic was increased. Further, the activity of biosynthesized AgNPs was investigated by Rajeshkumar et al. [122] by using a culture supernatant of *Enterococcus* sp. against pathogenic bacteria, fungi, and cancer cell lines. The AgNPs were spherical with a size range of 10–80 nm and it shows excellent enhanced antimicrobial activity than the commercial antibiotics. Generally, *Bacillus* sp.*, S. nematodiphila, B. subtilis,* and *Streptococcus* sp. were resistant to penicillin. However, in this study, the penicillin impregnated AgNPs showed significantly increased ZOI. These AgNPs also exhibited excellent enhanced fungicidal activity against all kinds of clinical isolates (*A. fumigatus, A. niger, A. flavus, C. albicans, Fusarium* sp.), among which maximum enhanced antifungal activity was noted against *Fusarium* sp. and *C. albicans*. In their study, they also found that the AgNPs were able to reduce the cell viability of the cancer cell line (Hep G2 and A549 cell line).

Recently, Singh et al. [123] produced spherical, 10 to 30-nm-sized AgNPs using a bacterial strain called *Weissella oryzae* DC6 that was isolated from mountain ginseng. The antimicrobial activity against pathogenic microorganisms including *C. albicans, V. parahaemolyticus, B. anthracis, S. aureus, B. cereus,* and *E. coli* was tested by measuring the ZOI and the results demonstrated that the AgNPs exhibited maximum antimicrobial activity against the tested microorganisms in the following manner: *S. aureus* followed by *C. albicans, B. cereus, V. parahaemolyticus, E. coli*, and lastly *B. anthracis.* In this study, it is shown that 5–6 μg concentrations of AgNPs are enough to inhibit the biofilm formation by *S. aureus* and *P. aeruginosa*. In another work, AgNPs were synthesized from endosymbiont *P. fluorescens* CA 417 inhabiting *Coffea arabica* L [124]. The TEM micrograph displayed the average size of nanoparticles to be between 5 and 50 nm with myriad shapes spherical, near to spherical, hexagonal, and triangular. The activity of synthesized nanoparticles was evaluated against phytopathogens such as *X. campestris*, *X. oryzae*, and *X. vesicatora,* and a significant result was observed. The synergistic effect of AgNPs in combination with antibiotic kanamycin resulted in increased fold dilution up to 58 % against *K. pneumoniae* (MTCC 7407) followed by *P. aeruginosa* (MTCC 7903), *B. subtilis* (MTCC 121), *S. aureus* (MTCC 7443) and *E. coli* (MTCC 7410).

A photo-irradiation-based method was developed for the synthesis of AgNPs by using *S. mesophila* MPKL 26 species for the first time [125]. TEM reveals that the AgNPs produced were spherical and sized in the range of 4–50 nm. The synthesized AgNPs show good antimicrobial activity against multidrug-resistant *S. aureus.* AuNPs and AgNPs were synthesized using *M. resistens* by Wang et al. [54]. The formation of gold nanoparticles is indicated by changing the color from yellow to crimson within 10 h whereas for AgNPs the color change from yellow to brown within 48 h. The synthesized gold and AgNPs have spherical shapes and sizes in the range of 10–20 nm. They were tested for antimicrobial activity against a range of pathogenic microorganisms which included *V. parahaemolyticus, S. enterica, S. aureus, B. anthracis, B. cereus, E. coli,* and *C. albicans* by measuring the ZOI and the results demonstrate that the pathogenic organisms are killed successfully by AgNPs, whereas, the gold nanoparticles have no influence on it. The results also show that as compared with antibiotics, AgNPs exhibit a clear and immense zone of inhibition around the disk.

Koilparambil et al. [126] synthesized AgNPs by using *E. coli* which is specified by a change in color of the reaction mixture from pale yellow to brown within 24 h of inoculation. The SEM images showed that the silver nanoparticles' shape was spherical with a size range from 5 to 25 nm in diameter. The antimicrobial efficacy of AgNPs (pellet and supernatant) was studied and it was found that the nanoparticles from the supernatant showed better activity. The MIC of AgNPs from the pellet was 10 μg mL^−1^, 50 μg mL^−1^, 20 μg mL^−1,^ and 10 μg mL^−1^ for *B. subtilis*, *S. typhi, K. pneumoniae,* and *V. cholerae* respectively. Gold and silver nanoparticles were also synthesized in different works by Singh et al. [127] by using *Sphingomonas koreensis* (*S. koreensis*) DC4 strain. FE-TEM results discovered the spherical shape of synthesized silver and gold NPs.The nanoparticle size range in the current study was from 30 to 50 nm. Nevertheless, the nanoparticles created with *S. koreensis* were not completely monodisperse. AgNPs showed MIC value against pathogenic strain in the range of 3–6.9 μg mL^−1^. The improvement of antibacterial activity of commercial antibiotics against pathogenic microorganisms including *S. aureus*, *V. parahaemolyticus, S. enterica, E. coli, B. anthracis,* and *B. cereus* was tested and the results revealed a significant increase in activity. The average maximum increase in fold area against antibiotic-resistant microorganisms; *E. coli, V. parahaemolyticus,* and *S. enterica*, was observed maximum for novobiocin (5.27 fold), followed by rifampicin (5.12 fold), lincomycin (4.88 fold), oleandomycin (4.66 fold), penicillin G (4.50 fold) and then vancomycin (4.34 fold).

Extracellular AgNPs with a distributed size predominantly between 25 and 45 nm were created using a *P. aeruginosa* strain from a reference culture collection, and TEM investigation revealed that they are spherical and pseudosperical in shape [128]. In this study, the antimicrobial activity was tested using clinical and reference strains of *S. epidermidis, E. coli, E. faecalis, P. mirabilis, S. aureus, A. baumannii, P. aeruginosa,* and *K. pneumoniae* with different concentrations of AgNPs (from 0.1 to 51.2 pM) using the conventional tube macrodilution method to determine Minimal inhibitory concentration (MIC) and MBC (Minimal bactericidal concentration) of the Ag-NPs. Minimal inhibitory concentration (MIC) is the lowest concentration of a chemical that prevents the growth of bacteria, whereas MBC is the lowest concentration of a chemical that results in bacteria death. The MIC measured between 0.4 and 6.4 pM at picomolar values. When compared to ciprofloxacin, AgNPs exhibit more growth and inhibition. In another study, AgNPs were produced after adding the purified exopolysaccharides (EPS) produced by *S. violaceus* MM72 to the aqueous silver nitrate solution [27]. The size of the nanoparticles ranges from 10 to 60 nm with 30 nm as an average having spherical shape. The antibacterial activity was observed against *E. coli* and *P. aeruginosa* with ZOI of 16 mm at 100 μg mL^−1^ concentration; whereas the *B. subtilis* and *S. aureus* showed ZOI of 10 and 4 mm at the same concentration. The MIC values were 6.15 and 11.6 μg mL^−1^ against *E. coli* and *P. aeruginosa*; whereas the *S. aureus* and *B. subtilis* arrested at 14.6 and 12.5 μg mL^−1^ respectively. Gram-negative bacteria *E. coli* and *P. aeruginosa* were more susceptible than the Gram-positive bacteria *B. subtilis* and *S. aureus*.

A halotolerant bacterium *B. endophyticus* SCU-L was used for the synthesis of AgNPs by Longzhan Gan et al. [129] and found that the AgNPs have a typical size of about 5.1 nm having a spherical shape. The ZOI for the nanoparticles against *E. coli* (13 mm), *S. typhi* (11 mm) *C. albicans* (15 mm), and *S. aureus* (19 mm) further revealed the broad-spectrum antimicrobial efficacy of silver nanoparticles toward pathogenic bacteria (both Gram-positive and Gram-negative) and one fungus strain. *Streptomyces xinghaiensis* (*S. xinghaiensis*) [130] OF1 strain was used for the synthesis of AgNPs with spherical and polydispersed nanoparticles in the size range of 5–20 nm. For *P. aeruginosa*, *C. albicans*, *M. furfur*, *B. subtilis*, *E. coli*, *K. pneumoniae* and *S. aureus*, the MIC values were 16, 32, 32, 64, 64, and 256 μg mL^−1^ respectively. AgNPs in combination with antimicrobial agent shows high synergistics effect against tested bacteria and fungi. Ampicillin/sulbactam, meropenem, chloramphenicol, and cefazolin in combination with AgNPs had the strongest cytotoxic effect.

**Fig. 13:**
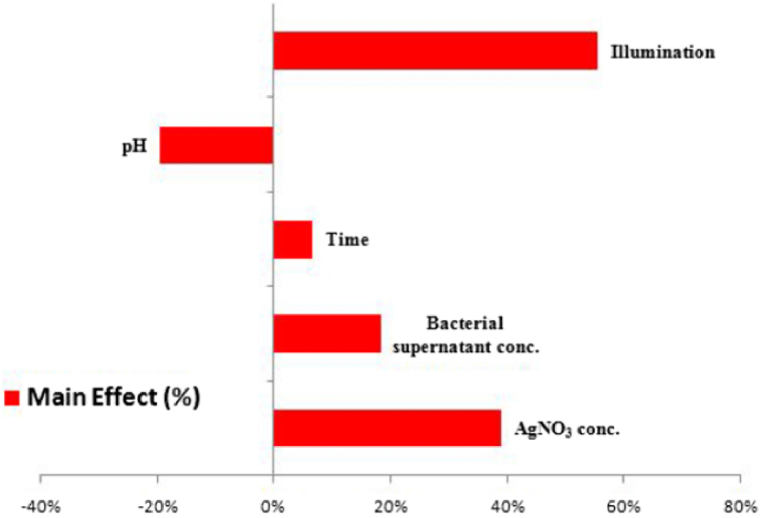
The main effects of the process variables on the biosynthesis of AgNPs using the supernatant of *L. adecarboxylata* THHM according to the Plackett–Burman experimental results. Reproduced from Ref. [134], (Creative Common Attribution 4.0 International License) Copyright 2022, Springer.

The difference between the antimicrobial action of crude AgNPs and calcined AgNPs at 200 ^°^C for 30 min was studied from the synthesized AgNPs using a strain *B. subtilis* KMS2-2 by extracellularly [131]. The size of the particles ranged from 18 to 100 nm and 49–153 nm before and after calcination, respectively, and were spherical. The antibacterial activities of crude and calcined AgNPs were tested against pathogenic bacteria such as *P. fluorescens* MTCC 1749, *P. mirabilis* MTCC 425, *E. coli* MTCC 1610, *B. cereus* and *S. aureus* MTCC 2940 by well diffusion method. Although the crude AgNPs have good inhibitory activity against tested pathogens, the calcined AgNPs do not show any inhibition. In a recent work, bacterial stem and root rot disease of sweet potato caused by *D. dadantii* was treated with AgNPs synthesized using cell-free culture supernatant of a bacterium *P. rhodesiae* and showed good antibacterial activity. The AgNPs had a diameter of 20–100 nm and were mainly uniform and spherical. The AgNPs displayed antibacterial properties against *D. dadantii* proliferation, swimming motion, biofilm development, and maceration of sweet potato tuber [132]. Recently, in 2022, *Streptomyces hirsutus* strain SNPGA-8 was reported for the synthesis of AgNPs with sizes ranging from 18.99 to 32.09 nm. The synthesized NPs showed antimicrobial activity against tested microbes such as *S. aureus, P. aeruginosa, E. faecalis, E. coli, C. albicans, A. alternata,* and *C. oxysporum.* The MIC and MBC were lowest for *P. aeruginosa* at 16 and 32 (μg mL^−1^), respectively. The highest antimicrobial activity was recorded against Gram-positive bacteria *E. feacalis* with ZOI of 21 mm at a volume of 100 μL AgNPs suspension, whereas aram-negative bacteria (*E. coli*) is more resistant with ZOI of 17 mm [133]. In another work, *Leclercia adecarboxylata* (*L. adecarboxylata*) THHM was also reported for the green synthesis of AgNPs extracellularly by Abdelmoneim et al. [134]. The authors examined the effect of different concentrations {10, 20, 40, 60, 80, and 100 % (v/v)} of the supernatant of the bacterial isolate and recorded the best concentration for the maximum production of silver nanoparticles. As the supernatant concentration of *L. adecarboxylata* THHM was increased from 10 to 20 %, the production of AgNPs also increased. If there is a further increase in the bacterial supernatant concentration greater than 20 % then it can cause a decrease in the production of AgNPs. The authors further studied five different parameters such as illumination, AgNO_3_ concentration, pH, bacterial supernatant concentration, and time that affect the biosynthesis of AgNPs as shown in Fig. 13. It was observed that parameters such as illumination, AgNO_3_ concentration, bacterial supernatant concentration, and time displayed a positive effect on the biosynthesis of AgNPs, while only pH showed a negative effect. In addition, the silver nanoparticles showed antimicrobial activities against several test pathogens such as *B. cereus, E. coli, S. aureus, V. cholera, P. aeruginosa, K. pneumoniae,* and *C. albicans.*

AgNPs produced using *Bacillus thuringiensis* (*B. thuringiensis*) were studied for their antibacterial and anti-biofilm properties by Khaleghi et al. [135]. AgNPs have a roughly spherical form and are 42 ± 7 nm in size, according to FE-SEM pictures. The antimicrobial effect of synthesized AgNPs was examined in six reference microorganisms such as *S. aureus, L. monocytogenes, K. pneumoniae, P. aeruginosa,* and *E. faecalis.* AgNPs have to be present at a minimum concentration of 6.25–25 g mL^−1^ to inhibit and kill pathogens. At a concentration of 6 g mL^−1^, more than 90 % of bacterial biofilm was destroyed. The characterization and antibacterial activity of the AgNPs synthesized using the *A. faecalis* GH3 cell extract were examined by Arastoo et al. [136]. According to their research, ampicillin combined with AgNPs had an antibacterial activity improvement of up to 162.5 % against *B. cereus* when compared to antibiotics alone. The bacterial test used were *E. coli, S. typhimurium, P. aeruginosa, K. pneumoniae, B. subtilis, S. aureus* and *B. cereus.* According to SEM data, AgNPs were spherical and ranged in size from 32 to 49 nm. Saied et al. [137] reported extracellular biosynthesis of AgNPs using *Cytobacillus firmus* (*C. firmus*) that showed inhibition against *S. aureus*, *P. aeruginosa*, *C. albicans, E. coli* and *E. feacalis*. Interestingly, the authors observed that biosynthesized AgNPs have an effect on Gram-positive than Gram-negative bacteria. At a ZOI of 16 mm, *C. albicans* was highly susceptible to the antifungal action.

The antimicrobial activity of AgNPs synthesized using exopolysaccharide of probiotic *Lactobacillus brevis* (*L. brevis*) MSR104 isolated from Chinese koumiss was excellent in a dose-dependent manner against both Gram-positive and Gram-negative bacteria [138]. SEM and TEM analysis revealed that the AgNPs have a spherical shape with an average size of 45 nm. The study also demonstrates that all the pathogenic microbes *S. aureus* and *E. coli* are greatly inhibited by 60 μL of 100 μg mL^−1^ biosynthesized AgNPs and they showed MIC values of 8.2 μg mL^−1^, 12.5 μg mL^−1^ against *E. coli* and *S. aureus*, respectively. A dextran-type exopolysaccharide (EPS) was utilized as a stabilizer agent in a recent study for the green synthesis of silver nanoparticles (AgNPs-Dex) from *Weissella cibaria* (*W. cibaria*) [139]. The antibacterial action of AgNPs-Dex against foodborne pathogenic bacteria *S. typhimurium, E. coli, Staph. aureus, B. cereus,* and *Yersinia enterocolitica* (*Y. enterocolitica*) were tested at 1 mg mL^−1^, 2.5 mg mL^−1,^ and 5 mg mL^−1^ concentrations and generally AgNPs-Dex were effective to all tested pathogenic bacteria. All fungi were inhibited by the application of AgNPs-Dex, except *B. cinerea*, when the antifungal activity of AgNPs-Dex was tested against *A. alternata*, *B. cinerea*, *A. niger*, *P. chrysogenum,* and *F. oxysporum* at 1–5 mg mL^−1^ AgNPs-Alt concentrations. TEM study revealed a homogeneous shape of AgNPs-Dex with a size of roughly 10 nm, and EDX analysis demonstrated the embedding of Ag ^+^ ions within dextran as the stabilizer agent. *L. plantarum* TA4 was also reported for the intracellular synthesis of spherical AgNPs. The DLS study discovered that the polydispersity index (PDI) value of synthesized AgNPs was 0.193, indicating the monodispersed characteristic of NPs. The AgNPs were found to stop the growth of tested microbes such as *S. epidermidis, E. coli, Salmonella* sp*.,* and *S. aureus* [140].

A bacterial leaf blight (BLB) disease caused by *Xanthomonas oryzae* pv*. oryzae* (Xoo) which reduces crop yield was found to be effectively controlled by employing AgNPs generated by *B. cereus* SZT1 [141]. The highest antibacterial activity was obtained in AgNP suspension at 20 g mL^−1^, which also had the biggest inhibition zone diameter (25.11 0.35 mm). The AgNPs at four different concentrations (5, 10, 15, and 20 μg mL^−1^) caused (38.85 %, 45.14 %, 57.71 %, and 91.42 %) reduction of Xoo at OD600 respectively. The outcome showed that organically produced AgNPs had excellent potential for preventing rice leaf blight caused by bacteria. SEM and TEM show that the AgNPs were spherical with particle sizes ranging from 18 to 39 nm. The synergistic antibacterial efficiency of Bacteriocin and AgNPs produced by probiotic *L. paracasei* against Multidrug multidrug-resistant bacteria was studied and it was found that the bioconjugate has high antibacterial efficiency. Although the bacteriocin was sensitive to MDR microorganisms, however, it may be degraded before it reaches the target. The bioconjugate bacteriocin-AgNPs have considerably improved cell membrane permeability and protein and DNA leakage. TEM micrograph shows that the shape of the particle is sphere-shaped and its size ranges from 8.33 to 26.17 nm [142].

AgNPs prepared from biosurfactant of *B. vallismortis* MDU6 strain exhibited its effectiveness against both Gram-negative and Gram-positive bacterial test microbes. It was most effective against *E. coli*, with a ZOI of 19 ± 2.5 mm, followed by *S. aureus* (16 ± 2 mm), *L. monocytogenes* (17 ± 1.5 mm), and *B. subtilis* (12 ± 2 mm). No cytotoxicity against primary mouse liver cell lines was shown by the AgNPs. SEM images showed that the AgNPs were sphere-shaped [143]. In 2023, extracellular biosynthesis of quasi-spherical AgNPs using cyanobacterium *N. carneum* was revealed by Borah et al. [28]. They observed, for all concentrations of AgNPs, a higher inhibition against Gram-negative bacteria (*E. coli, P. aeruginosa*) than against Gram-positive *S. aureus.* This can be attributed to the fact that for the Gram-positive bacteria, the thick peptidoglycan layer hindered nanoparticle entrance through the cell wall, resulting in a lower antibacterial effect.

In another work by Iqtedar et al. [29] AgNPs were synthesized irregular shape AgNPs by using *B. mojavensis* BTCB15 and optimized to study the antimicrobial activity. At concentrations ranging from 0.5 to 2.5 mg mL^−1^, the AgNPs exhibited antibacterial efficacy against Gram-negative bacteria such as *E. coli* BTCB03, *K. pneumoniae* BTCB04*,* and *Acinetobacter* sp. BTCB05 and *P. aeruginosa* BTCB01, but not against Gram-positive bacteria such as *S. aureus* BTCB02. This discovery demonstrated that AgNPs are highly efficient against Gram-negative bacteria. The addition of surfactant Tween 20, and metal ion K_2_SO_4_, reduces the size of the particles to about 104 % with a typical size of 2.3 nm and this increases the antibacterial action against multidrug-resistant pathogens.

The enhancement of the activity of antibiotics by AgNPs was shown by Gurunathan [144] in his work. Supernatants from *B. cereus* cultures with a size of 10 nm were used to make AgNPs. The antibacterial activity of the selected antibiotics with AgNPs was tested against two bacterial test strains, viz. *S. mutans* and *E. fergusonii*. The activity of the six antibiotics tested with AgNPs, ampicillin (10 g mL^−1^), chloramphenicol (30 g mL^−1^), erythromycin (15 g mL^−1^), gentamicin (10 g mL^−1^), tetracycline (30 g mL^−1^), and vancomycin (30 g mL^−1^), was considerable against both Gram-negative and Gram-positive bacteria. The highest increase in *E. fergusonii* was reported for gentamycin (p <0.05), while in the case of *S. mutans*, the highest increase was observed for vancomycin (p <0.05). In another work [145], AgNPs were synthesized by using cell-free supernatant of mutant *B. licheniformis* M09 which are sphere-shaped. AgNPs synthesized using *B. licheniformis* M09 are smaller in size and they most likely have improved cell wall penetration capacities which enhances their ability to have a cidal impact on Gram-negative bacteria.

Silver nanoparticles prepared by *B. amyloliquefaciens* were examined for antibacterial and antifungal activity against Gram-negative, Gram-positive, and fungus strains. In-vitro studies demonstrated that AgNPs have potent antifungal properties against phytopathogenic *F. oxysporium* (33 mm IZD) and *F. solani* (26 mm IZD) [146]. In another study, the antimicrobial efficacy of the AgNPs produced by using *B. subtilis* was examined against five strains of multidrug-resistant microorganisms including *S. epidermidis, S. aureus* (MRSA), *E. coli, K. pneumoniae,* and *C. albicans* tested as yeast [147]. Interestingly, the synthesized AgNPs showed a higher inhibition rate than the standard antibacterial drug (Streptomycin) and antifungal drug Fluconazole. According to TEM micrographs, the particles were sphere-shaped or almost rounded in form, and their size ranged from 3 to 20 nm.

Ahmed et al. [148] studied AgNP synthesis by a silver-resistant *B. safensis* TEN12 strain isolated from metal-contaminated soil and taxonomically identified using 16S rRNA gene sequencing. The role of capping proteins and alcohols in the stability of AgNPs was established by Fourier transform infrared (FTIR) spectroscopy. SEM and TEM investigation showed that the biogenic AgNPs were spherical, with particle sizes ranging from 22.77 to 45.98 nm. The antibacterial activity of AgNPs was tested against *S. aureus* and *E. coli*, and the diameters of ZOI were found to be (13.16, 16.92, 17.01, and 20.35 mm) against *S. aureus* at four varying concentrations of AgNPs (5, 10, 15 and 20 g mL^−1^) respectively, whereas for *E. coli* at a concentration of (5, 10, 15 and 20 g mL^−1^) respectively. In another investigation, the green synthesized AgNPs using *P. eburnea* MAHUQ-39 were tested for their antimicrobial activity against multidrug-resistant pathogenic microbes (*E. coli, S. aureus,* and *P. aeruginosa*). From their study, it was revealed that there is a clear ZOI around the discs saturated with synthesized AgNPs. The antibacterial activity of the synthesized AgNPs was highest against drug-resistant *P. aeruginosa*, followed by *S. aureus* and *E. coli.* The MICs of green-produced AgNPs for *P. aeruginosa* and *S. aureus* were 6.25 g mL^−1^ and 100 g mL^−1^, respectively, showing that the synthesized AgNPs greatly reduced the proliferation of these two pathogens [149].

For the first time, the use of *Desertifilum* sp. for the biosynthesis of AgNPs has been reported by Hamida et al. [150]. Five bacterial strains of Gram-positive and Gram-negative bacteria (*P. aeruginosa* ATCC 27853, *B. cercus* ATCC 10876, *B. cereu*s ATCC 10876, *B. subtilis* ATCC 6633, *S. flexneri* and *S. enterica*) were evaluated and the ZOI diameter for *S. flexneri* was 22.7 ± 0.3, 16.7 ± 0.3 for *S. enterica*. In 2022, *Lactiplantibacillus plantarum* (*L. plantarum*) was reported for the synthesis of spherical shape AgNPs [151] that successfully demonstrated outstanding inhibition against urinary tract infecting (UTI) pathogens ranging from 14.42 to 22.18 mm against *E. coli, P. mirabilis, P. aeruginosa, K. pneumoniae, S. aureus,* and *E. faecalis.* Delightfully, the AgNPs also demonstrated greater antibacterial activity as compared to standard antibiotic drug *gentamycin* as can be seen in Table 3. The AgNPs also displayed excellent synergistic effects in combination with several antibiotics for example chloramphenicol, streptomycin, vancomycin, and gentamycin. A very high 75 % fold increase in ZOI was seen against *P. aeruginosa* for Vancomycin-AgNPs combined compared to Vancomycin alone, presenting a high synergistic effect of AgNPs and Vancomycin.

**Table 3:** Antibacterial activity of *L. plantarum* promoted AgNPs against UTI pathogens against standard antibiotic gentamycin.

Sl. no	Pathogens	Zone of inhibition	
		AgNPs	Standard antibiotic, gentamycin
1.	*E. coli*	22.18 ± 2.09	20.67 ± 2.03
2.	*S. aureus*	14.42 ± 0.90	12.37 ± 0.82
3.	*P. mirabilis*	20.08 ± 1.14	19.37 ± 1.05
4.	*P. aeruginosa*	19.77 ± 1.19	17.90 ± 1.14
5.	*K. pneumoniae*	16.57 ± 1.82	14.07 ± 1.41
6.	*E. faecalis*	19.50 ± 1.94	17.40 ± 1.15

**Table 4:** Biosynthesis of AgNPs using different bacteria and cyanobacteria.

Sl. No	Bacteria	Mode of synthesis	Shape/Size	Test microorganism	Ref.
1.	*Corynebacterium glutamicum*	Extracellular	Spherical/15 nm	*B. subtilis, S. aureus, S. enterica, P. aueroginosa, E. coli, K. pneumoniae, B. flexus*	[48]
2.	*Pseudomonas putida MVP2*	Extracellular	Spherical/5–16 nm	*S. aureus, B. cereus, E. coli, P. aeruginosa, H. pylori*	[55]
3.	*Macrococcus bovicus*	Extracellular	Spherical/5.4–21.5 nm	*C. albicans, E. coli, S. aureus*	[49]
4.	*Rhodococcus* spp.	Intracellular	Spherical/10–30 nm	*S. aureus, P. vulgaris, K. pneumoniae, E. faecalis, P. aeruginosa, E. coli*	[114]
5.	*Streptomyces strains*	Intracellular	Spherical/1.17–13.3	*S. aureus, S. typhi, P. aeruginosa, K. pneumoniae, E. coli, P. vulgaris*	[115]
6.	*Pseudomonas veronii*	Extracellular	Spherical/5–50 nm	*E. coli, S. aureus, P. aeruginosa, X. axonopodis* pv*. malvacearum, X. oryzae* pv*. oryzae, R. solanacearum, K. pneumonia, B. subtilis*	[50]
7.	*Lactobacillus acidophilus*	Extracellular	Spherical/4–50 nm	*K. pneumonia*	[51]
8.	*Streptomyces* spp.	Extracellular	Spherical/198–595 nm	*K. pneumoniae, B. subtilis, S. flexneri, E. aerogenes, S. typhi-B, P. vulgaris, S. epidermidis, S. typhimurium*	[116]
9.	*Bacillus safensis*	Extracellular	Spherical/5–30 nm	*E. coli*	[52]
10.	*Brevibacterium frigoritolerans*	Extracellular	Spherical/97 nm	*E. coli, S. enterica, B. anthracis, B. cereus, C. albicans, Vibrio parahaemolyticus* (*V. parahaemolyticus*)	[117]
11.	*Bacillus safensis*	Extracellular	Spherical/5–95 nm	*S. aureus, Klebsiella granulomatis* (*K. granulomatis*)*, P. aeruginosa*	[53]
12.	*Delftia* sp.	Extracellular	Spherical/9.8 nm	*Candida strains*	[24]
13.	*Bacillus methylotrophicus*	Extracellular	Spherical/10–30 nm	*C. albicans, S. enterica, V. parahaemolyticus, E. coli*	[118]
14.	*Actinobacter* sp.	Extracellular	Spherical/13.2 nm	*E. coli, B. subtilis, S. aureus*	[119]
15.	*Actinobacter* sp.	Extracellular	Spherical/4–45 nm	*B. subtilis, E. coli, P. aeruginosa, S. aureus, K. pneumoniae, P. mirabilis, S. infantis*	[120]
16.	*Pilimelia columellifera*	Extracellular	Spherical/12.7–15.9 nm	*S.aureus, K. pneumoniae, P. aeruginosa, Enterobacter, B. subtilis, P. aeruginosa, K. pneumoniae, E. coli*	[121]
17.	*Enterococcus* sp.	Extracellular	Spherical/10–80 nm	*Bacillus* sp*., K. pneumoniae, S. nematodiphila, Streptococcus* sp*., A. niger, A. fumigatus, A. flavus, B. subtilis, K. planticola, C. albicans, Fusarium* sp.	[122]
18.	*Weissella oryzae*	Intracellular	Spherical/10–30 nm	*B. anthracis,S. aureus, E. coli, C. albicans, B. cereus, V. parahaemolyticus,*	[123]
19.	*Pseudomonas fluorescens*	Extracellular	Spherical/5–50 nm	*E. coli, S. aureus, K. pneumonae, P. aeruginosa, B. subtilis*	[124]
20.	*Sinomonas mesophila*	Extracellular	Spherical/4–50 nm	*S. aureus*	[125]
21.	*Microbacterium resistens*	Extracellular	Spherical/10–20 nm	*S. enterica, V. parahaemolytic, S. aureus, B. anthracis, B. cereus, C. albicans, E. coli*	[54]
22.	*E. coli*	Extracellular	Spherical/118 nm	*S. typhi, V. cholerae, B. subtilis, K. pneumoniae*	[126]
23.	*Sporosarcina koreensis*	Extracellular	Spherical/102 nm	*V. parahaemolyticus, S. aureus, B. cereus, B. anthracis, E. coli, S. enterica*	[127]
24.	*Pseudomonas aeruginosa*	Extracellular	Spherical/25–45 nm	*S. aureus, P. mirabilis, Acinetobacter baumannii* (*A. baumannii*)*, E. coli, P. aeruginosa, K. pneumonia, E. faecalis*	[128]
25.	*Streptomyces violaceus* MM72	Intracellular	Spherical/10 nm–100 nm	*S. aureus, B.subtilis, P. aeruginosa, E. coli,*	[27]
26.	*Bacillus endophyticus SCU-L*	Extracellular	Spherical/5.1 nm	*S. typhi, C. albicans, E. coli, S. aureus.*	[129]
27.	*Streptomyces xinghaiensis*	Extracellular	Spherical/5–20 nm	*P. aeruginosa, S. aureus, E. coli, B.subtilis,* yeasts *viz., C. albicans, M. furfur*	[130]
28.	*Bacillus subtilis* KMS2-2	Extracellular	Spherical/18–100 nm	*Proteus mirabilis* (*P. mirabilis*)*,P. fluorescens, B. cereus, E. coli, S. aureus*	[131]
29.	*Pseudomonas rhodesiae*	Extracellular	Spherical/20–100 nm	*Dickeya dadantii* (*D. dadantii*)	[132]
30.	*Streptomyces hirsutus* strain SNPGA-8	Extracellular	Spherical/18.99–32.09 nm	*P. aeruginosa, C. glabrata, E. faecalis, C. albicans, Alternaria alternata* (*A. alternate*)*, F. oxysporum, S. aureus, E. coli*	[133]
31.	*Leclercia adecarboxylata* THHM	Extracellular	Spherical/3.48–39.02 nm	*B. cereus, V. cholera, K. pneumoniae, C. albicans, E. coli, P. aeruginosa, S. aureus*	[134]
32.	*Bacillus thuringiensis*	Extracellular	Spherical/42 nm	*S. aureus, E. coli, L. monocytogenes, K. pneumoniae, E. faecalis, P. aeruginosa*	[135]
33.	*Alcaligenes faecalis*	Extracellular	Spherical/32–49 nm	*K. pneumoniae, E. coli, S. aureus, B. subtilis, S. typhimurium, P. aeruginosa, B. cereus*	[136]
34.	*Cytobacillus firmus*	Extracellular	Spherical/30–60 nm	*S. aureus, P. aeruginosa, Enterococcus faecalis* (*E. feacalis*), *C. albicans, E. coli*	[137]
35.	*Lactobacillus brevis*	Extracellular	Spherical/45 nm	*S. aureus, E. coli*	[138]
36.	*Lactobacillus Plantarum TA4*	Intracellular	Spherical/14.0 nm	*S. aureus, S. epidermidis, E. coli, Salmonella* sp.	[140]
37.	*Weissella cibaria*	Extracellular	Spherical/10 nm	*S. typhimurium, Penicillium chrysogenum* (*P. chrysogenum*)	[139]
38.	*Bacillus cereus*	Extracellular	Spherical/18–39 nm	*Xanthomonas oryzae* pv*. oryzae*	[141]
39.	*Lactobacillus paracasei*	Extracellular	Spherical/8.33–26.17 nm	*S. aureus, Streptococcus pyogenes* (*S. pyogenes*)*, K. pneumonia, E. coli, P. mirabilis, P. aeruginosa, P. vulgaris*	[142]
40.	*Bacillus vallismortis*	Intracellular	Spherical/44–70 nm	*B. subtilis, L. monocytogenes, S. aureus*	[143]
41.	*Nostoc carneum*	Extracellular	Quasi-spherical/4–22 nm	*P. aeruginosa, K. pneumonia, E. coli, S. aureus*	[28].
42.	*Bacillus mojavensis*	Extracellular	Irregular/2.3 nm	*S. aureus, K. pneumonia, E. coli, Acinetobacter* sp*., P. aeruginosa*	[29]
43.	*Bacillus cereus*	Extracellular	Spherical/2–16 nm	*E. fergusonii, S. mutans*	[144]
44.	*Bacillus licheniformis*	Extracellular	Spherical/10–30 nm	*B. subtilis* subsp*. spizizenii, E. coli, S. aureus, P. aeruginosa*	[145]
45.	*Bacillus amyloliquefaciens*	Extracellular	Spherical/1.23–10.80 nm	*S. aureus, E. coli, P. aeruginosa, F. solani, F. oxysporum, B. subtilis*	[146]
46.	*Bacillus subtilis*	Intracellular	Spherical/3–20 nm	*S. aureus, S. epidermidis, K. pneumoniae, E. coli, C. albicans*	[147]
47.	*Bacillus safensis*	Extracellular	Spherical/22.77–45.98 nm	*S. aureus, E. coli*	[148]
48.	*Pseudoduganella eburnea*	Extracellular	Spherical/8–24 nm	*S. aureus, P. aeruginosa, E. coli*	[149]
49.	*Desertifilum* sp*. [cyanobacteria]*	Intracellular	Spherical/4.5–26 nm	*P. aeruginosa, S. enterica, B. subtilis, S. flexneri, B. cereus*	[150]
50.	*Lactobacillus plantarum*	Extracellular	Spherical/40–50 nm	*K. pneumoniae, P. mirabilis, P. aeruginosa, S. aureus, E. faecalis, E. coli*	[151]
51.	*Pantoea ananatis*	Extracellular	Spherical/8.06–91.32 nm	*C. albicans* (ATCC 10231), *E. coli* (ATCC 10536), *S. aureus* subsp*. aureus* (ATCC 11632), *B. cereus* (ATCC 10876), *S. pneumoniae* (ATCC 700677)	[152]
52.	*Cytobacillus kochii*	Extracellular	Spherical/4–44 nm	*S. typhi, P. aeruginosa, E. coli, A. baumannii, K. pneumoniae, S. aureus,*	[153]
53.	*Paracoccus* sp*.* Arc7*-*R13	Extracellular	Spherical, ellipsoidal/2–25 nm	*B. subtilis, P. aeruginosa, E. coli, S. aureus*	[154]
54.	*Oscillatoria limnetica [cyanobacteria]*	Extracellular	Quasi-spherical/3.30–17.97 nm.	*E. coli, B. cereus*	[155]
55.	*Bacillus* spp*. (Bacillus pumilus, B. persicus* and *B. licheniformis)*	Extracellular	Triangular, spherical hexagonal/80, 92, and 77 nm, respectively	*C. albicans,Bean Yellow Mosaic Virus, K. pneumoniae, P. aeruginosa, E. coli, S. epidermidis, S. sonnei, A. flavus*	[156]
56.	*Bhargavaea indica*	Extracellular	Pentagon, spherical, icosahedron, hexagonal, Nanobar, truncated triangle, and triangular shapes/30–100 nm	*S. enterica, S. aureus, B. cereus, E. coli, C. albicans, V. parahaemolyticus, B. anthracis*	[157]
57.	*Bacillus subtilis*	Extracellular	Spherical, hexagonal, and irregular; 20 nm	*P. aeruginosa, S. typhi, C. albicans, S. aureus, rotavirus*, *E. coli, B. cereus*	[158]
58.	*Saccharopolyspora hirsuta*	Extracellular	Star, spherical/10–30 nm	*S. pyogenes, P. aeruginosa, S. typhi, C. albicans, K. pneumoniae, S. aureus*	[159]
59.	*Pseudomonas* sp*. THG-LS1.4*	Extracellular	Irregular/10–40 nm	*E. coli, S. aureus, C. tropicalis, P. aeruginosa B. cereus, V. parahaemolyticus, S. enterica.*	[160]
60.	*Bacillus zanthoxyli GBE11*	Extracellular	Polydisperse/3.68–31.60 nm	*S. typhi enterica* (ATCC25922)*, E. coli* DH5α*, B. subtilis* (WB800), *S. aureus* (ATCC6538P)*, P. aeruginosa* (ATCC27853)	[161]

**Fig. 14:**
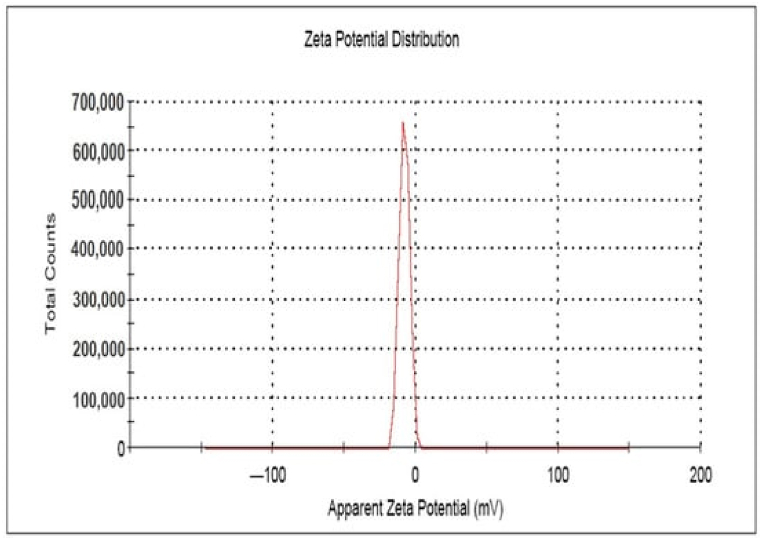
Zeta potential of the biosynthesized AgNPs. Reproduced from Ref. [152], (Creative Common CC BY License), Copyright 2018, MDPI.

In another study, *P. ananatis* was used for the synthesis of sphere-shaped AgNPs with a typical size of 8.06–91.32 nm. The AgNPs were tested against *S. aureus* subsp*. aureus* (ATCC 11632), *E. coli* (ATCC 10536), *P. aeruginosa* (ATCC 10145), *B. cereus* (ATCC 10876), *C. albicans* (ATCC 10231) and multidrug-resistant *S. pneumoniae* (ATCC 700677). Stability plays an important role in the application of NPs in the field of biomedical. The mean of the zeta potential value was found as −7.48 ± 0.64 mV, which strongly supports the formation of moderately stable colloidal AgNPs (Fig. 14) [152].

A spherical AgNP was recently reported to have been synthesized using *C. kochii* Strain SW6. Interestingly, the *C. kochii* promoted green AgNPs (CkCE-AgNPs) showed much higher inhibition compared to chemically synthesized AgNPs (CAgNPs) towards the tested microbes. Accordingly, a relatively low MIC of 6.5, 12.5, 12.5, 6.5, 12.5, and 12.5 μg mL^−1^ was found for CkCE-AgNPs) whereas, MIC for CAgNPs was 50, 100, 100, 50, 100, and 5,0 μg mL^−1^ against *A. baumannii, S. typhi, E. coli, K. pneumoniae, S. aureus,* and *P. aeruginosa,* respectively [153]. Even though spherical AgNPs were mainly obtained by biosynthesis using bacteria, other shapes such as ellipsoidal, quasi-spherical, triangular, hexagonal, and pentagonal shapes were also produced. Zheng et al. [154] describe the synthesis and antibacterial activity of AgNPs using an Arctic anti-oxidative bacterium *Paracoccus* sp. Arc7-R13. The shape of the AgNPs was spherical and ellipsoidal with sizes ranging from 2 to 25 nm as revealed by TEM analysis. Antimicrobial tests against Gram-negative *P. aeruginosa, E. c,oli,* and Gram-positive *S. aureus*, *B. subtilis* show that the AgNPs had strong activity.

Hamouda et al. [155] used an aqueous extract of *Oscillatoria limnetica* (*O. limnetica*) fresh biomass for the green synthesis of quasi-spherical ANPs. TEM images revealed a quasi-sphericAgNPNPs morphology with a size range of 3.30–17.97 nm. In their studies, the appearance of clear inhibitory zones confirmed the complete growth inhibition of either *E. coli* or *B. cereus*. The diameter of the inhibition zone was 22 mm against *E. coli* and 20 mm against *B. cereus*. The synergistic effect of Cefaxone conjugated AgNPs showed a 26 mm inhibition zone diameter against *E. coli* and Tetracycline conjugated AgNPs showed a 24 mm inhibition zone diameter against *B. cereus*. In the recent past, Essam et al. [156] worked on the synthesis of AgNPs using *Bacillus* species namely, *B. persicus, B. pumilus,* and *B. licheniformis* and their size-dependent antimicrobial activities against bean yellow mosaic virus and human pathogen. The typical size of AgNPs produced by *B. pumilus* (NPs-1), *B. persicus* (NPs-2), and *B. licheniformis* (NPs-3) was 80, 92 and 77 nm, respectively. The results of their study revealed that NPs-3 had the smallest MICs and MBCs values when compared to NPs-1 and NPs-2. This fact can be attributed to the size of NPs-3, which is the smallest of all AgNPs evaluated in this study.

**Fig. 15:**
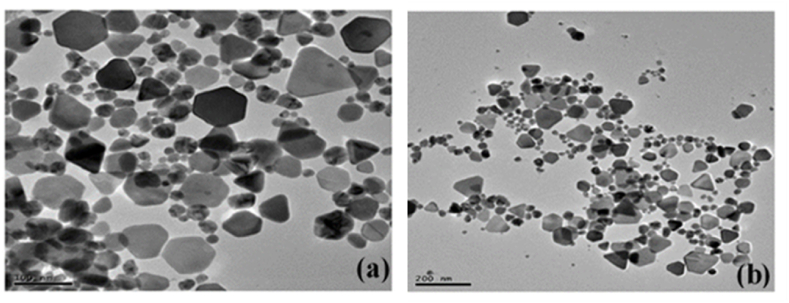
UV–vis spectra of *B. indica* DC1 culture supernatant after treatment with (1 mM) AgNO_3_ (a). Anisotropic AgNPs produced by *B. indica* DC1, 100, and 200 nm in a TEM image (b). Reproduced from Ref. [157] (Creative Common Attribution License), Copyright 2015, Hindawi.

Singh et al. [157] examined the synergistic effect of AgNPs generated by *B. indica*. According to their findings, the AgNPs obtained have nano bar, spherical, hexagonal, truncated triangle, icosahedron and triangular nanoparticles with sizes ranging from 30 to 100 nm, as illustrated in Fig. 15a–b. The biosynthesized AgNPs exhibited antimicrobial activity against a range of pathogenic microorganisms such as *S. enterica*, *V. parahaemolyticus*, *S. aureus*, *B. anthracis*, *C. albicans*, *B. cereus* and *E. coli* and the mean diameter of the zone of inhibition were 23 ± 0.5, 22 ± 1, 18 ± 0.8, 16 ± 0.6, 15 ± 0.4, 14 ± 1, 10 ± 1. The examined microorganism became more susceptible due to the combination of antibiotics (oleandomycin, novobiocin, penicillin G, vancomycin, lincomycin, and rifampicin) and biosynthesized AgNPs.

In another work, characterization and the activity of AgNPs synthesized by using *B. subtilis* were reported. The AgNPs were polycrystalline in shape, including spherical, hexagonal, and irregular geometries, and an average diameter of 20 nm. The antibacterial activity was tested on *E. coli, B. cereus, S. aureus, P. aeruginos,* and *S. typhi* and it showed that the MIC was found to be 41–43 μg mL^−1^ except for *P. aeruginosa* where MIC was 169 μg mL^−1^. In their studies, the cytotoxic effect on the liver (HepG-2) and lung (A549) cancer cell lines and also on African green monkey kidney epithelial cell line (MA104) showed IC_50_ of 212.5, 6.4, and 78.9 mg mL^−1^ respectively. The AgNPs studied also exhibit strong antiviral activity against rotavirus infection. The AgNPs were integrated into various textile and wound dressings and antimicrobial activity is still seen after five cycles of washing [158]. In another investigation, star-shaped AgNPs were biosynthesized using a novel strain of *S. hirsuta*. The star shape and the size range distribution from 10 to 30 nm with polydispersity was confirmed by TEM. The antibacterial activity of the produced nanoparticles was tested, and at 30 g/disc, the strongest inhibitory activity was obtained against *S. aureus* (13 mm), *K. pneumonia* (13 mm), *S. pyogenes* (12 mm), *S. typhi* (12 mm), *P. aeruginosa* (13 mm) and *C. albicans* (11 mm) [159].

Singh et al. [160] prepared AgNPs using the *Pseudomonas* sp. (THG-LS1.4) strain. The synthesized AgNPs were crystalline and irregular in shape with the size varying from 10 to 40 nm. They have tested the biosynthesized AgNPs for antimicrobial activity against *B. cereus, S. aureus, C. tropicalis, E. coli, P. aeruginosa, V. parahaemolyticus,* and *S. enterica* and the average zone of inhibition are 16.5 ± 0.6, 11.5 ± 0.7, 16.0 ± 0.2, 14.5 ± 0.5, 13.0 ± 0.3, 16.5 ± 0.5 and 11.5 ± 0.8 respectively. In their investigation, they showed that the AgNPs increased the activity of commercial antibiotics to a great extent. The average increase in fold area of the antibiotics against antibiotics bacteria (*E. coli*, *P. aeruginosa,* and *S. enterica*) was the greatest for erythromycin (15.0 ± 1.1), followed by novobiocin (15.0 ± 0.8), penicillin G (14.0 ± 1.3), oleandomycin (14.0 ± 1.5), lincomycin (11.0 ± 1.7) and then vancomycin (11.0 ± 2.3). They hypothesized that the bonding reaction between antibiotic and nanosilver may be what is causing the rise in synergistic impact. Furthermore, at a concentration of 10 g mL^−1^, AgNPs prevent biofilm formation in *P. aeruginosa* and *S. aureus*.

Recently, Li et al. [161] reported the green polydisperse AgNPs synthesis using an extracellular extract of *Bacillus zanthoxyli* (*B. zanthoxyli*) GBE11. To get the most stable AgNPs, they optimized the AgNPs synthesis process parameters such as AgNO_3_ concentration, reaction temperature, and reaction periods. Based on the UV–vis spectra, while optimizing the silver nitrate concentration, the SPR peak of AgNPs improved marginally from 2.61485 (1 mmolL^−1^) to 3.35395 (3 mmolL^−1^) as the AgNO_3_ concentration increased. Moreover, the wave peak of AgNPs synthesized from 4 mmolL^−1^ onwards was disordered and unstable, and the absorption peak was quite broad. This could be because the reducing agents in the bacterial extract are unable to support the reduction of excess Ag^+^ in the reaction solution, where AgNPs formed with unreacted AgNO_3_ lead to NP aggregation, as previously reported [152]. Low stability of the produced AgNPs will cause them to combine and form larger NPs, which will have less antibacterial activity. Hence, 3 mmolL^−1^ solutions are the optimized silver nitrate concentration. Likewise, a reaction temperature of 35 °C and a time of 72 h were found to be the optimized conditions to produce stable AgNPs. The synthesized AgNPs further showed antimicrobial activity against *P. aeruginosa* (ATCC27853), *S. aureus* (ATCC6538P)*, B. subtilis* (WB800)*, S. typhi enterica* (ATCC25922), and *E. coli* (DH5α).

## 4 Factors affecting the biosynthesis of silver nanoparticles

Several factors such as phytochemicals in microbes, the concentration of silver nitrate solution, reaction time, pH, and reaction temperature have pronounced effects on the successful biosynthesis of AgNPs and are discussed below.

### 4.1 Phytochemicals in microbial extracts

The phytochemicals (biomolecules) present in the microbes (proteins, nucleic acid, polysaccharides, lipids, primary and secondary metabolites) that can act as a reducing agent are solely responsible for the biochemical reduction of Ag^+^ ions to metallic Ag° nanoparticles. Hence, information about the biochemicals present in microbes is very important for their potential application in the synthesis of AgNPs [162]. Rajoka et al. [138] determined the total amount of carbohydrates which is responsible for the reduction of Ag^+^ ions to metallic Ag° nanoparticles by the phenol-sulfuric acid method and found that the *L. brevis* MSR104 used to produce AgNPs contain a total carbohydrate content of 65.3 ± 1.5 %. Chowdhury et al. [71] also used the phytopathogenic fungus *M. phaseolina* for AgNPs and found that the proteins present in the cell-free extract act both as reducing and capping agents.

### 4.2 Concentration of the silver nitrate solution

During the biosynthesis of AgNPs, increasing the concentration of AgNO_3_ led to the formation of a higher amount of AgNPs up to the level where all the AgNO_3_ salt is consumed such that all Ag ^+^ ions are reduced to metallic Ag°. The reduction of Ag^+^ ions to metallic Ag° is usually observed using UV–Vis spectroscopy, where a characteristic peak for AgNPs is observed at 300–500 nm [47,114]. An equilibrium will be reached at a point when all the AgNO_3_ is consumed. Beyond this point, adding more AgNO_3_ solution will result in the formation of unstable AgNPs as can be seen in UV–Vis spectroscopy which might be owing to the agglomeration of silver nanoparticles with Ag ions [161]. A concentration of 1 mM or 2 mM AgNO_3_ was usually used with different volumes to the filtrate of incubated microbes. A concentration of 3 mM [27,49,120] and as high as 4 mM was also used [146,154]. Therefore, a good balance between the AgNO_3_ concentration and the amount of reducing agent present in the microbial extract needs consideration.

### 4.3 pH

pH plays a very important role in the stability and size of the biosynthesized AgNPs. Most often at acidic pH, the size of the AgNPs is large and at alkaline pH, the size of the silver nanoparticle is usually small [48,50,116]. The nucleation mechanism for the formation of AgNPs is known to be delayed at acidic pH and hence resulted in the formation of large-size, agglomerated NPs [40] whereas, in alkaline pH, fast nucleation process occurred due to accessibility of OH^−^, thus faster reaction happens and a large number of small size particles are formed in a shorter time [48,119]. Nonetheless, high pH potentially promotes undesirable precipitation of AgOH/AgO_2_ [163]. Zeta potential values more than +30 mV and less than −30 mV are regarded as stable zeta potential values for NPs. Owing to electron-fixed revulsion, NP aggregation is difficult for elements with a zeta-balancing potential [90]. The surface charge stability of the produced silver nanoparticle was determined by zeta potential analysis. The zeta potential of AgNPs indicated a slightly negative charge at a lower pH value of 3 (−3.2 mV). The zeta potential of AgNPs decreased monotonically from - 12.1 mV at pH 7.0 to −24.4 mV at pH 11.0, indicating that more stable NPs were formed.

### 4.4 Reaction time

The reaction time is the amount of time necessary for silver nanoparticle synthesis to occur from the time the reactant is added to the beaker. AgNO_3_ was added to the supernatant extract of the cultured microbes and then the mixture was allowed to react for reduction of Ag ions to metallic AgNPs, mostly for a few hrs to 24 h [48,51], but sometimes the reaction period also took a longer time, as high as 48 h [54,119,132]. AgNP formation can be easily visualized from color changes from generally light yellow (microbes extract and silver nitrate mixture) to brownish color indicating the reduction of silver nitrate to metallic AgNPs [47,54,126]. As the size of the AgNPs increases with time due to the continuous nucleation process [164], as observed by a red-shift in UV–Vis spectrometer data, careful analysis of the reaction mixture to obtain a stable small size, yet highly stable AgNPs, is of utmost important.

### 4.5 Reaction temperature

From a sustainability and economic point of view, biosynthesis of AgNPs under room temperature (RT) or near RT is desirable. Most reported literature synthesized AgNPs at RT and relatively low temperatures [94]. Li et al. [161] reported that 35 °C is the optimized reaction temperature where the most stable AgNPs are observed, above which unstable NPs were obtained as shown by a chaotic absorption band in UV–Vis spectroscopic analysis. However, the AgNPs produced at elevated temperatures of 47 °C–52 °C showed agglomeration of AgNPs [163]. Additionally, Lade et al. showed that increasing the reaction temperature resulted in a quick decrease in the rate of Ag^+^ ions and subsequent homogenous nucleation of silver nuclei, allowing the formation of tiny-size AgNPs. It has also been found that when the temperature of the reaction mixture increases, the rate of nanoparticle production slows while the stability rises. Moreover, AgNPs produced at higher temperatures have smaller nanoparticle diameters [165].

## 5 Factors affecting the antimicrobial activity of AgNPs

Antibacterial activity is influenced mainly by different factors such as size, shape, pH, concentration, and surface chemistry which are discussed below.

### 5.1 Size of the AgNPs

One of the numerous additional factors that affected the antibacterial activity of the AgNPs was their size. In most observations, AgNPs having smaller sizes have higher activity since NPs having smaller dimensions are associated with easier bacterial cell wall penetration, and more intense destruction by reactive oxygen species (ROS) accumulation [164,166]. Lu et al. [167] investigated the impact of size on the antibacterial activity of AgNPs against the bacteria that cause periodontal and caries disorders. By chemically reducing AgNPs with polyvinylpyrrolidone (PVP), AgNPs of 5, 15, and 55 nm were synthesized, and their antibacterial activity against *F. nucleatum*, *S. sanguis*, *E. coli*, *S. mitis*, *S. mutans,* and *A. actinomycetemcomitans* was assessed. It was found that 5 nm nanoparticles had a more potent antibacterial effect, with minimum inhibitory concentrations (MIC) ranging between 25 and 50 g mL^−1^ for all of the tested pathogens, except the assayed strain of *E. coli*, whose MIC value was 6 mL^−1^. In another work to elucidate the importance of smaller-size NPs, Roelofs et al. [168] demonstrated that small and medium-sized AgNPs greatly impact mitochondrial electron transport, autophagy, phagocytosis, organelle integrity, etc. leading to cell death. Essam et al. [156] also observed that *Bacillus licheniformis* (*B. licheniformis*) mediated AgNPs with average size 77 nm revealed the lowest MICs and MBCs values and also higher antimicrobial activities Bean Yellow Mosaic Virus and human pathogens compared to those with bigger nanoparticle size 80 and 92 nm produced by *Bacillus pumilus* (*B. pumilus*) and *B. persicus* (*Bacillus pumilus*), respectively. These observations revealed that smaller AgNPs are more efficient compared to bigger size NPs as antimicrobials. Hence, the production of extremely small-size AgNPs is an outstanding challenge at this time for their potential applications in biomedical fields.

### 5.2 Shape of the AgNPs

The shape of the NPs also plays an important role in their antibacterial activity. Within the same size ranges, owing to the presence of sharp edges and vertexes, the triangular shape has the highest activity while the spherical shape has the least activity [169,170]. Similarly, among the different shapes, when the size of particles was similar, penta- and hexagonal AgNPs showed higher antimicrobial activity than spherical and rectangular NPs [164]. The antibacterial efficacy of variously shaped AgNPs against *E. coli* was examined by Hong et al. Both time-response tests and dose-response tests were undertaken. Stronger antibacterial action is demonstrated by a longer delay time [171]. At an AgNP concentration of 0.15 gmL^−1^, 6 h was sufficient to kill all the bacterial cells in nanocubes, while nanospheres and nanowires did not appear to lose viability. AgNP-sp completely inactivated the bacteria after 6 h at a dosage of 1.5 gm L^−1^. Yet, after 6 h, there were still numerous live bacterial cells for silver nanowires (AgNP-Ws).

### 5.3 pH

In general, bacterial membranes are negatively charged whereas the mammalian cells are of negligible net charge, and, in most cases acidic microenvironment is observed in bacterial infection sites. These factors were taken into consideration when creating pH-induced charge switchable AgNPs that would have less cytotoxicity towards mammalian cells in neutral solutions and more toxicity versus bacteria in an acidic milieu. In a pioneering work, Qiao et al. [172] observed a pH-dependent MIC and MBC for AgNPs against microbes such as *S. aureus* and *E. coli*. The MIC and MBC values of the AgNPs at pH 4.7 against *E. coli* and *S. aureus* were much lower than those of the AgNPs at pH 7.4. Under physiological conditions, the carboxyl groups deprotonated, resulting in AgNPs with zwitterions and good cytocompatibilities. At the same time, the protonation of carboxyl groups in an acidic fluid results in the oxidation of metallic AgNPs to positively charged Ag^+^ ions, which is the main active species that leads to desired microbial cell death. A similar research by Dat et al. [173] revealed that the bactericidal activity of the nanocomposite reduces as pH rises. The pH environment with the best bactericidal action among the three was 5.6. More H^+^ ions and soluble oxygen are present in the acidic environment, which causes AgNPs to oxidize and become Ag^+^ ions. As a result, the low pH speeds up the release of Ag^+^, increasing Ag/antibacterial rGO's action.

### 5.4 Concentration and contact time of AgNPs

AgNP concentration and its contact time with the targeted microbial cells both have an impact on how effective they are against microbial cells. In general, AgNP concentration rises when the antibacterial activity increases. Liao et al. [174] examined the antibacterial activity of AgNPs by MIC and MBC in a concentration- and time-dependent method on clinical isolates of resistant *P. aeruginosa*. In a recent work by Fouda et al. [84], the antimicrobial activity of AgNPs decreased with decreasing concentrations For instance, the largest inhibition zones were observed for AgNPs at a concentration of 100 ppm with a diameter of 18.7 ± 0.5, 17.7 ± 0.6, 20.7 ± 0.6, and 20.8 ± 0.3 mm for *B. subtilis*, *S. aureus*, *P. aeruginosa* and *E. coli*, respectively, whereas at 50 ppm, the formed inhibition zones were decreased to 15.3 ± 0.5, 14.7 ± 0.6, 18.5 ± 0.5, 18.8 ± 0.3, 14.7 ± 0.6, 15.3 ± 1.2, 13.3 ± 0.6, and 13.8 ± 0.3 mm for *B. subtilis*, *S. aureus*, *P. aeruginosa*, *E. coli*, *C. albicans*., respectively.

In similar work, *S. cerevisiae* (Baker's yeast) mediated AgNPs were also found to show dose-dependent antimicrobial activity. For the maximum AgNPs dose (100 μg mL^−1^), the pathogens *S. aureus*, *C. albicans*, *B. subtilis*, *P. aeruginosa,* and *E. coli* showed a high level of inhibitory activity with inhibition zones of 21 ± 2.1, 20.9 ± 0.7, 19 ± 0.8, 18 ± 1.4, and 15.6 ± 1.6 mm, respectively. Whereas at a lower concentration of 50 μg mL^−1^ of AgNPs, it demonstrated lower antimicrobial properties against the tested microorganisms *C. albicans*, *B. subtilis*, *P. aeruginosa*, *S. aureus* and *E. coli* with inhibition value was 18 ± 0.8, 16.3 ± 1.2, 15.3 ± 1.2, 15.3 ± 1.4, and 13.1 ± 1.4 mm, respectively [85].

### 5.5 Surface charge and coating (capping)

**Fig. 16:**
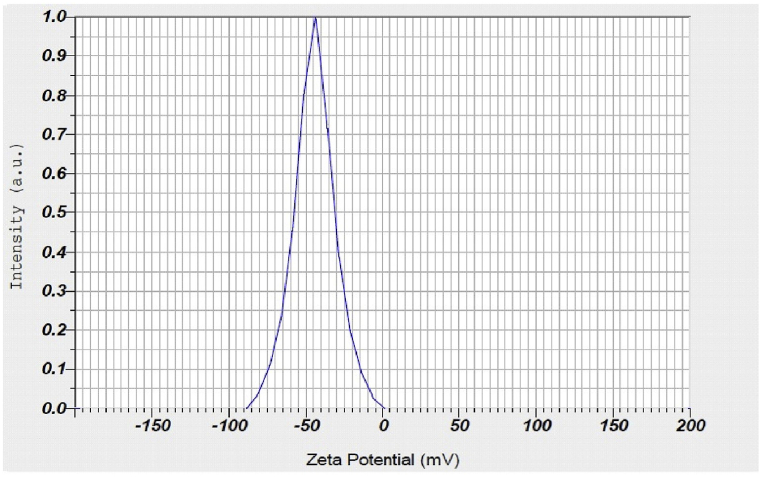
Zeta Potential (mV) of *P. hornemannii* mediated AgNPs. Reproduced with permission from Ref. [90], Copyright 2019, Elsevier.

The attachment of AgNPs to the microbial cell wall initiates the interaction between AgNPs and microorganisms. Negatively charged microbial cell membranes and positively or less negatively charged AgNPs are electrostatically attracted to one another, which disrupts membrane permeability and respiratory functions through membrane depolarization, which in turn disrupts the integrity of the cell and results in cell death [30]. The biosynthesized AgNPs having high surface charge density will be highly unstable and they would tend to aggregate and form bigger particles with lower antibacterial activity. The principal factors governing the stability of AgNPs are the charge and the capping/coating. To avoid agglomeration due to the repulsive interactions between NPs, NPs are thought to be stable if their zeta potential values are greater than +30 mV or more negative than 30 Mv [90,175]. Zeta potential standards are deemed to be balanced when they are greater than +30 mV and greater than −30 mV, however when the value of zeta potential is zero, the elements are considered to be less balanced. In their investigation, Saeb et al. [175] produced AgNPs using *P. putida* (S5 isolate), *C. sedlakii* (S11P isolate), and *E. hermannii* (SHE isolates) and used the zeta potential value to confirm the stability of AgNPs produced using different strains. Similarly, Fatima et al. also produced AgNPs with zeta potential - 44.5 mV which proved the formation of highly stable NPs from *P. hornemannii* (Fig. 16) [90].

According to research, coating nanoparticles with polymers or organic substances results in NPs that are low or nontoxic to mammalian cells while maintaining their lethal effects on tested bacteria. For instance, coating AgNPs with chitosan has demonstrated a strong inhibitory effect against *S. aureus*, *S. typhimurium,* and *P. aeruginosa*, resulting in up to 95 % fewer colonies after 4 h of contact [176]. It has also been proved that the AgNPs surface-coated with negatively charged bovine serum albumin (BSA) showed higher potential than positively charged chitosan (Chit) or negatively charged polyvinylpyrrolidone (PVP) coated NPs [168]. Biological molecules play dual roles in the biosynthesis of NPs, acting as an *in situ* reducing and capping agent as well as reducing the metal salts. This capping has various advantages, including reducing NP agglomeration, lowering toxicity, and enhancing antibacterial activity [177,178]. A synergistic effect between metal NPs and the capped biomolecules may be observed, as in the case of amoxicillin-coated gold NPs [179], if these coating agents themselves exhibit antibacterial activity.

Jain et al. [65] reported the synthesis of protein-capped AgNPs in an aqueous solvent system. They further proceeded to prepare bare AgNPs by comparing the antibacterial efficacy of protein-capped and bare AgNPs. The findings showed that AgNPs with varied antibacterial capabilities were more efficient than protein-capped AgNPs against the tested Gram-negative and Gram-positive bacterial species, and it was concluded that the presence of protein shells on AgNPs can lessen their bactericidal effects. In the current research, the release of Ag^+^ ions from protein-capped and bare AgNPs which was mainly believed to cause microbial cell membrane damage was estimated using ICP-AES, and was found that the dissolved Ag^+^ ion concentration for protein-capped and bare AgNPs was measured as 15.8 and 27.5 μg L^−1^, respectively. This indicated that the presence of a protein shell acts as a barrier preventing the release of Ag ions [25] from the protein-coated AgNPs, thereby reducing its antibacterial potential compared to bare AgNPs. Hence, although protein and biomolecules attached to AgNPs, have a positive effect on the stability of NPs, controlling the degree of coating of AgNPs by any protein and (or) biomolecules to obtain the most active antimicrobial AgNPs without compensating the stability of AgNPs is very crucial.

## 6 Molecular resistance mechanism of microbes against AgNPs

Gram-negative bacteria have evolved several defenses against the toxicity of silver ions (Ag^+^). The region between the outer and inner membranes of the bacterial cell wall, known as the periplasm, is where one of the key processes for limiting silver ions occurs. This stops silver ions from entering the cytoplasm, where they may otherwise impair crucial cellular functions [180]. Staehlin et al. reported that the first discovered silver resistance plasmid (pMG101) of the Gram-negative *Salmonella typhimurium* was thought to have its sil cluster after the removal of the pco genes from the island consisting of copper homeostasis gene clusters. SilA, SilB, SilC, and SilE (augmenting SilCBA transporter without a counterpart in the Cus system) are primary proteins for silver resistance [180]. Lok et al. have reported that the copper export system also contributes to the resistance mechanism of AgNP. In an *E. coli* strain with resistance to silver that was developed through exposure to higher concentrations of silver, they observed the activation of chromosomal genes responsible for producing copper resistance proteins CusF and CusB (and likely CusC and CusA as well). Additionally, a single change in a nucleotide within the cusS gene (analogous to silS) was identified [181].

The structural arrangement of the plasmid-based gene cluster known as “pco” in *E. coli* closely resembles that of the chromosomal “cop” operon cluster, except for the presence of the pcoE gene. Interestingly, this pcoE gene does not have counterparts in other copper resistance determinants but is analogous to the silE gene found in the soil system. PcoE performs exceptionally well as a metal-binding molecule in the periplasmic region because of its high affinity for both copper and silver ions. This enables it to efficiently sequester both Cu^+^ and Ag^+^ ions before the primary pco system proteins are activated [182]. Another method used by bacteria, including those resistant to silver, to defend themselves against stressors such as the presence of metallic cations like silver ions (Ag^+^), is biofilm development. Extracellular polymeric substances (EPS) serve as a matrix for the complex and organized populations of microorganisms known as biofilms, which stick to surfaces. The encapsulated microbial cells benefit from this biofilm matrix resilience and protection [183]. Indeed, multiple studies explore how bacterial biofilms respond to stress caused by silver, particularly in the context of silver nanoparticles (AgNPs). Aeromonas punctata has been reported to create EPS and subsequently coat the silver nanoparticles (AgNPs) with this layer of EPS in response to exposure to AgNPs. Most likely, this procedure acts as a safeguard to lessen the damaging effects of silver. Similar results were also obtained with Micrococcus luteus, *S. aureus,* and *E. coli* [184].

## 7 Comparative analysis of microbes-mediated synthesis of AgNPs with other nanoparticles

In this study, a comparison was conducted involving silver nanoparticles (AgNPs) alongside three other types of nanoparticles: iron oxide nanoparticles (Fe_3_O_4_ NPs) [185], gold nanoparticles (AuNPs) [186], and zinc oxide nanoparticles (ZnO NPs) [187] as shown in Table 5. We tried to compare the synthesis procedure, their shape and sizes, their anti-bacterial properties, and their application in various fields. However, it should be noted that the size of the nanoparticle varies from microbes to microbes. So basically here we have given a general size range of different nanoparticles.

**Table 5:** Comparative analysis of microbes mediated synthesis of AgNPs and other nanoparticles.

Nanoparticles	AgNPs	AuNPs [186]	Fe_3_O_4_ NPs [185]	ZnO NPs [187]
Synthesis	Various microorganisms are used as reducing and stabilizing agents in the synthesis of AgNPs.	Microorganisms are utilized to synthesize AuNPs with distinct shapes and sizes.	In the case of Fe_3_O_4_ nanoparticles, microorganisms are employed to reduce iron ions and synthesize iron oxide nanoparticles.	ZnO NPs are biologically synthesized utilizing physiologically active compounds from plants and microorganisms such as bacteria, fungi, and yeast.
Size and shape	Mostly spherical in shape and size ranging from 8.33 to 26.17 nm.	Mostly spherical but some are rod shape and size ranging between 80 and 45 nm.	Exhibit various shapes like spherical, rod-like, and complex geometries and size ranges from 5 to 50 nm.	The sizes vary in shape depending on the synthesis method. Exhibits different shapes (rod-like and isometric) and a broad size distribution range of 30–200 nm.
Antibacterial properties	AgNPs possess exceptional antimicrobial properties, making them suitable for medical, environmental, and industrial applications.	AuNPs exhibit excellent biocompatibility and stability.	Fe_3_O_4_ NPs possess superparamagnetism, enabling their use in MRI and targeted drug delivery.	Due to their small size, large surface area, composition, and morphology, enable them to interact effectively with bacterial cell surfaces, penetrate the cell interior, and execute bactericidal actions through distinct mechanisms. It possesses remarkable antimicrobial properties, particularly against bacteria and fungi.
Applications	They are used in wound healing, drug delivery, and water purification.	They find applications in drug delivery, biosensing, and imaging due to their unique optical properties.	They have applications in medical imaging and cancer therapy.	ZnO NPs have photocatalytic properties, making them suitable for sunscreens, wound healing, and water purification.

## 8 Challenges

Silver nanoparticles (AgNPs) have received significant attention because of their antimicrobial properties and potential uses in a variety of industries, including healthcare, electronics, and textiles. However, there are concerns regarding the toxicity and environmental impact of silver nanoparticles in living terrestrial or aquatic organisms.

Numerous studies have been conducted to understand the potential toxicity of AgNPs. These studies have revealed that AgNPs can have toxic effects on various organisms and ecosystems [188]. Historically, the toxicity of AgNPs on traditional targets of exogenous substances has been underestimated, with some sensitive systems such as reproductive and nervous systems requiring attention in toxicity assessments. Recent developments have highlighted the need for specific toxicity tests for nanomaterials, including AgNPs, to establish a comprehensive understanding of their potential health and environmental risks. Furthermore, the increasing use of consumer products containing AgNPs may result in higher levels of environmental silver, which can have detrimental effects [189,190]. Regulatory control over the use and disposal of AgNP-enhanced products is currently lagging due to insufficient assessment of the toxicology of AgNPs and their rate of release into the environment. Therefore, there is a critical need for thorough research and monitoring to assess the toxicity and environmental impact of AgNPs to establish effective regulations and safeguard human health and the environment.

Pham et al. [191]. have recently reported the impact of increased synthesis and application of AgNPs on aquatic systems. Generally, in an aqueous environment, AgNPs tend to dissolve to Ag^+^ under the presence of oxygen. Thus, the presence of both AgNPs and Ag^+^ in the environment, and the hazardous impacts of AgNPs on algal cells were contributed by AgNPs and their released Ag^+^. Tortella et al. examine the toxicity of AgNPs and the possible tolerance mechanisms that organisms may employ to mitigate their deleterious effects. Furthermore, the article emphasizes the ongoing debate surrounding the environmental impact of AgNPs and the need for further research to fully understand their effects on live terrestrial and aquatic organisms. It also highlights the importance of accurate characterization and assessment methods to ensure the safe use of AgNPs in various applications. The presence of AgNPs in aquatic habitats has resulted in harmful effects on aquatic creatures as well as environmental degradation [192]. Long-term studies have shown that AgNPs can accumulate in various organs and tissues following chronic exposure. Accumulation in organs such as the liver, kidneys, and brain has been reported. Bioaccumulation of AgNPs over time may lead to prolonged exposure to silver ions, which could potentially result in toxic effects. Studies have shown that AgNPs can induce DNA damage and chromosomal aberrations in exposed cells [193]. Yu et al. discuss the increasing use of silver nanoparticles (AgNPs) in various industries and the potential risks they pose to health and the environment. Furthermore, the article highlights the importance of conducting additional in vivo toxicity studies to determine the exact mechanism of toxicity of AgNPs and predict their health effects on humans. Understanding the potential risks associated with AgNPs is crucial for ensuring the safe and responsible use of these nanoparticles in various applications [194]. Hence, significant efforts have been made in recent years to overcome these issues to make the synthesis of AgNPs more efficient and cost-effective. The biological synthesis also known as the “green synthesis” method is widely used by scientists to synthesize silver nanoparticles which involves the use of microorganisms (fungi, bacteria), micro and macro algae/plants as nano-factories to reduce the cost and also reduces the generation of hazardous waste materials. Since the synthesis of these AgNPs using different microbes has become a demanding topic of research in this review, we tried to highlight different microbes' mediated biosynthesis of silver nanoparticles and their enhanced antimicrobial activities.

## 9 Conclusion and future trends

In conclusion, the microbes mediated intra- and extracellular biosynthesis of AgNPs using eco-friendly and non-toxic methods have undergone substantial research in recent decades. In the biosynthesis of AgNPs, several factors need to be optimized to obtain the most stable, robust, and highly efficient antimicrobial NPs. The phytochemicals in the microbial extracts worked as a reducing agent and a capping agent throughout the biosynthesis to stabilize the generated AgNPs. The green silver nanoparticles showed high antimicrobial activity against several pathogenic microbes including many multidrug-resistant microbes [120,122,127,128]. Delightfully, many of the bio-AgNPs exhibit higher antimicrobial activity as compared to other biosynthesized NPs such as ZnO NPs [86], and Au NPs [54]. In addition, the antimicrobial activity of AgNPs can be greatly enhanced by their synergetic interaction with commercial antibiotic drugs. This review also revealed the beneficial effects of commercial antibiotics and biosynthesized AgNPs in combination against pathogenic microorganisms [24,36,40,120,136,157,160]. Penicillin, Ampicillin, and Novobiocin coated with AgNPs exhibit excellent inhibition zones against *Bacillus* sp.*, B. subtilis, S. nematodiphila,* and *Streptococcus* sp*.* [122]. The enhancement of commercial antibiotics, novobiocin, lincomycin, vancomycin, penicillin G, erythromycin, and oleandomycin by AgNPs was also tested against bacteria including *P. aeruginosa, S. enterica,* and *E. coli* and the zone of inhibition increased. Although a clear concept is unknown, some studies suggested that the increase in this activity of commercial antibiotics is due to the bonding reaction between antibiotics and AgNPs. Through chelation, the antibiotic with active sites such as hydroxyl and amido group reacts with AgNPs [160].

One of the greatest challenges to human society at present is the increasing number of multidrug-resistant microbes. Hence, many researchers are looking for an alternative to currently available antibiotic drugs. In this line, of great interest, many green AgNPs are known to possess higher inhibition against many multidrug-resistant microbes than commercially available antibiotic drugs such as penicillin and oleandomycin [54], fluconazole [64], streptomycin [147], gentamycin [151]. As mentioned earlier, in general, a Gram-positive bacterium (eg. *B. subtilis* and *S. aureus*) consistently exhibited lower susceptibility against the AgNPs than a Gram-negative bacterium (eg. *P. aeruginosa* and *E. coli*) [[27], [28], [29]]. This is because the strong peptidoglycan layer of Gram-positive bacteria hindered nanoparticle entrance through the cell wall, resulting in a lower antibacterial effect. Nonetheless, in some cases, compared to Gram-negative bacteria, Gram-positive bacteria demonstrated greater sensitivity against AgNPs [85,133,137]. Hence, it is highly desirable to have a better insight into the interaction between the AgNPs and the bacterial cell walls leading to cell death. Therefore, knowing the intricacy of the research on the biosynthesis and antimicrobial activity of AgNPs, several factors contributing to their biosynthesis, and their resultant size, shape, pH, time, and surface chemistry-dependent antimicrobial efficacies of the biosynthesized AgNPs are worth considering during AgNPs synthesis.

The potential applications of AgNPs synthesized from microbial sources may continue to expand, opening up new avenues for innovative solutions in biotechnology, medicine, and environmental sustainability. Silver nanoparticles can be employed in strong disinfectants to prevent microbial infections due to their many potent properties [195]. A recent breakthrough is the use of silver nanoparticles in magnetic disinfection systems to treat waterborne diseases due to their numerous robust actions in powerful disinfectants for preventing microbiological infections [196]. Magnetic oxide is one of the active components used in magnetic disinfection [197]. It has been shown that using silver nanoparticles as a sorbent and driving force for removing ecological contaminants has proven to be effective. Nanosilver-based disinfection solutions have the potential to be useful in decontaminating surfaces, other types of equipment, and computers, perhaps leading to the creation of nanosilver-based consumer products in the future. Silver-based nanocomposites can be environmentally gainful, and they might be utilized in the improvement of unique product applications.

## Authorship contribution statement

Each author has made substantial contributions to the development and writing of this article.

## Data availability statement

No data was used for the research described in the article.

## CRediT authorship contribution statement

**Chhangte Vanlalveni:** Writing – review & editing, Writing – original draft, Visualization, Resources, Investigation, Conceptualization. **Vanlalhruaii Ralte:** Resources, Investigation, Conceptualization. **Hlawncheu Zohmingliana:** Writing – review & editing, Resources. **Shikhasmita Das:** Writing – review & editing, Resources. **Jasha Momo H. Anal:** Visualization, Resources, Conceptualization. **Samuel Lallianrawna:** Visualization, Resources, Investigation. **Samuel Lalthazuala Rokhum:** Writing – review & editing, Writing – original draft, Supervision, Resources, Conceptualization.

## Declaration of competing interest

The authors declare the following financial interests/personal relationships which may be considered as potential competing interests:Dr Samuel Lalthazuala Rokhum reports administrative support was provided by 10.13039/501100015990National Institute of Technology Silchar Department of Chemistry. Dr Samuel Lalthazuala Rokhum reports a relationship with National Institute of Technology Silchar Department of Chemistry that includes: employment. The corresponding author is the Editorial Advisory Board member for Heliyon in the energy section. If there are other authors, they declare that they have no known competing financial interests or personal relationships that could have appeared to influence the work reported in this paper.
